# Vascularized human skin assembloids model compartmental skin organization and immune responsiveness in vitro

**DOI:** 10.1038/s12276-026-01840-x

**Published:** 2026-10-01

**Authors:** Min-Ji Kim, Seunghee Lee, Kyung-Sun Kang

**Affiliations:** 1 Adult Stem Cell Research Center, College of Veterinary Medicine, Seoul National University, Seoul, 08826 Republic of Korea; 2 Stem Cell and Regenerative Bioengineering Institute, Global R&D Center, Kangstem Biotech Co., Ltd., Geumcheon-gu, Seoul, Republic of Korea

**Keywords:** Stem-cell differentiation, Stem-cell biotechnology, Experimental models of disease, Stem-cell research

## Abstract

Allergic skin diseases, including atopic and contact dermatitis, are prevalent inflammatory disorders imposing clinical and socioeconomic burdens. Their pathogenesis involves impaired barrier function, genetic susceptibility and immune dysregulation, yet mechanistic insights remain limited by the lack of physiologically relevant human in vitro models. Conventional human skin organoids capture aspects of epidermal stratification but generally lack vascular components, limiting tissue maintenance and physiological relevance. To overcome these limitations, vascularized skin assembloids (VSAs) are generated by combining human induced pluripotent stem cell-derived skin organoids with blood vessel organoids under air–liquid interface conditions. This modular assembly supports the formation of key skin-like structural features, including a multilayered epidermis, organized dermal matrix and interconnected vascular-like networks associated with improved barrier maturation and dermal–vascular organization, while providing an accessible platform for epidermal allergen exposure and inflammatory analysis in a vascularized skin-like tissue context. Incorporation of immune cells establishes an immune-integrated and immune-responsive microenvironment that enables modelling of allergen-induced inflammation. Upon exposure to house dust mite extract, VSAs exhibit inflammatory remodelling with atopic dermatitis-relevant features, characterized by barrier disruption, keratinocyte stress responses, immune-cell infiltration and induction of type 2 mediators (IL-4, IL-13, TSLP, CCL17, CCL26). Treatment with clinically used atopic dermatitis therapeutics upadacitinib (JAK1 inhibitor) and dupilumab (IL-4Rα antagonist) improves barrier-associated features and suppresses STAT6-associated inflammatory signalling, showing responses consistent with their known mechanisms of action. Collectively, these findings support VSAs as a human-relevant, vascularized and immune-integrated skin assembloid model for mechanistic studies and preclinical evaluation of pharmacological responses in inflammatory skin diseases.

## Vascularized Skin Assembloids Enhance Allergy Research Insights

The study explores the development of vascularized skin assembloids (VSAs) using human induced pluripotent stem cells to address the limitations of existing skin models, which lack vascular and immune components. Researchers integrated human induced pluripotent stem cell-derived skin organoids and blood vessel organoids under air–liquid interface conditions to create VSAs with a stratified epidermis, fibroblast-rich dermis and interconnected endothelial networks. These VSAs supported immune co-culture and enabled allergen exposure, allowing for the analysis of inflammatory responses. The results showed that VSAs could model features of atopic dermatitis, including inflammatory remodelling and pharmacological modulation of JAK–STAT signalling. The study highlights the potential of VSAs as a platform for disease modelling and preclinical pharmacological evaluation, with future directions including the incorporation of microfluidic systems to enhance vascular maturity and the integration of patient-specific immune components for personalized medicine applications.

This summary was initially drafted using artificial intelligence, then revised and fact-checked by the author.

## Introduction

As the primary interface with the external environment, the skin maintains homeostasis through coordinated barrier, immune and sensory functions^1,2^. Structurally, it comprises a stratified epidermis, fibroblast-rich dermis and a dynamic vascular network that regulates nutrient transport, thermoregulation and immune-cell trafficking^3,4^. Owing to this structural and functional complexity, human skin has become a central focus in biomedical, cosmetic and pharmaceutical research, serving as both a biological interface and a translational testbed for therapeutic discovery^5,6^.

Over the past decades, multiple in vitro strategies have been developed to emulate human skin architecture and function. Two-dimensional keratinocyte monolayers and reconstructed three-dimensional cultures have been widely used for studies of epidermal differentiation and barrier physiology, whereas engineered human skin equivalents have enabled pharmacological testing and toxicological screening^7–9^. However, these systems lack key physiological features, including vasculature and immune-associated components, which are important for modelling inflammatory and vascular-associated skin diseases. Although animal models provide systemic complexity, interspecies differences, ethical considerations and limited scalability constrain their translational relevance^10^. Recent advances in tissue engineering, such as microfluidic skin-on-chip and bioprinting platforms, have partially addressed these challenges, yet still fall short in fully capturing the multicellular organization, dynamic signalling and tissue–immune interactions of native human skin^11,12^. In particular, the absence of vascular-associated components can restrict nutrient exchange, immune-cell trafficking and inflammatory crosstalk, thereby limiting long-term tissue maintenance and physiological modelling.

Human induced pluripotent stem cell (hiPSC) technology has opened up new opportunities for modelling organogenesis through the self-organization of multiple cell types. Skin organoids (SkOs) derived from hiPSCs recapitulate epidermal and dermal differentiation and generate appendages such as hair follicles, melanocytes and neurons^13,14^. Despite these advances, SkOs exhibit notable limitations compared with native human skin. They often display inverted polarity, in which the epidermis is positioned internally and the dermis externally, and frequently undergo ectopic cartilage differentiation^14,15^. These abnormalities compromise physiological fidelity and standardization, hindering their scalability for high-throughput applications such as disease modelling or drug screening. Moreover, the lack of vascular and immune-associated components remains a major constraint, as endothelial and immune networks are important for establishing microenvironmental gradients, mediating inflammatory responses and evaluating pharmacological responses.

Several recent approaches have attempted to overcome these limitations. Transient activation of WNT signalling during organoid development suppresses ectopic cartilage formation and promotes the generation of large, correctly oriented skin organoids suitable for modelling solar UV-induced damage and repair^16^. However, these models remain avascular and lack immune integration, which restricts their use for studying complex inflammation. In parallel, advances in the generation of hiPSC-derived blood vessel organoids (BVOs) have yielded self-assembled endothelial networks with lumina and mural support cells capable of forming perfusable vascular structures. Although these systems represent major progress towards physiologically relevant vascular complexity^15,17^, a modular and experimentally accessible skin model that enables controlled integration of epithelial, vascular and immune components, epidermal-side allergen stimulation and downstream pharmacological testing remains limited. The concept of assembloids, which modularly integrate lineage-specific organoids to reconstruct tissue–tissue interfaces, offers a promising strategy for overcoming these barriers^18^. Assembloids have recently been applied to model complex inter-organ interactions, including vascularized tumour systems and brain–immune interfaces, thereby enabling functional readouts such as immune infiltration and therapeutic response^19,20^. Inspired by these developments, we sought to create a human skin assembloid system that incorporates vascular and immune components while preserving experimental control over component assembly, tissue accessibility and inflammatory perturbation. In this context, the modular assembloid strategy is not simply a method for combining tissue components, but provides a controllable platform in which separately generated SkOs and BVOs can be assembled, matured under air–liquid interface conditions, exposed to epidermal allergens and subsequently analysed for coordinated epithelial, vascular and immune-associated responses.

In this study, we established vascularized skin assembloids (VSAs) by modularly integrating hiPSC-derived SkOs and BVOs under air–liquid interface culture. VSAs displayed compartmental organization comprising a stratified epidermis, a fibroblast-rich dermal compartment, hair follicle- and neural-associated elements, and interconnected endothelial networks with luminal structures. Unlike conventional organoid models, VSAs supported immune co-culture and provided an experimentally accessible architecture for epidermal-side allergen exposure, enabling analysis of vascular-associated inflammatory responses and pharmacological modulation. Functionally, VSAs modelled selected features of house dust mite (HDM)-induced inflammatory remodelling relevant to atopic dermatitis and responded to pharmacological modulation of JAK–STAT signalling, supporting their use as a structurally complex, vascularized and immune-integrated human skin assembloid model. Collectively, these findings position VSAs as an advanced platform for mechanistic dissection, disease modelling and preclinical pharmacological evaluation in human inflammatory skin biology.

## Materials and methods

### Human induced pluripotent stem cell culture

This study was carried out in accordance with the approved guidelines of the Seoul National University Institutional Review Board (IRB-No.2504/003-001). The hiPSC line CMC003 was obtained from the National Stem Cell Bank of Korea. HiPSCs were maintained in StemFlex (Gibco, USA) on vitronectin (Gibco)-coated dishes, and passaged with ReLeSR reagent (STEMCELL Technologies, Canada).

### Generation of induced pluripotent stem cell-derived human skin organoids

Based on previous reports^13,16^, iPSCs were differentiated into SkOs. Briefly, on day 0, a total of 1200 dissociated iPSCs/well were seeded in StemFlex with 20 μM ROCK inhibitor Y-27632 (Tocris, UK) in an ultra-low attachment 96-well plate (Corning, USA) to generate uniform embryoid bodies (EBs). When the size of the EBs became 250–300 μm, the EBs were transferred into individual new ultra-low attachment 96-well plates in Essential 6-based differentiation medium containing 2% Matrigel (Corning), 10 μM SB431542 (Tocris), 4 ng ml^−1^ bFGF (R&D Systems, USA), and 2.5 ng ml^−1^ BMP4 (Peprotech, USA) to initiate non-neural ectoderm induction. After 3 days of culture in non-neural ectoderm induction medium, 200 ng ml^−1^ LDN (Tocris), 50 ng ml^−1^ bFGF, and 15µM CHIR99021 (Tocris) were added at 25 μl per well to induce cranial neural crest cell formation. On day 6 of differentiation, 75 μl of fresh E6 medium was added. Half of the medium was changed on days 8 and 10. On day 12, to induce self-assembly of the epidermis, all organoids were transferred into ultra-low-attachment six-well plates and placed on an orbital shaker (SPL Life Science, Republic of Korea) in 3 ml of skin maturation medium containing a 1:1 mixture of advanced DMEM-F12 and Neurobasal medium (Gibco) supplemented with 1X N-2 supplement (Gibco), 0.5X B-27 without vitamin A supplement (Gibco), 2-mercaptoethanol (Sigma-Aldrich), GlutaMAX (Gibco) and Primocin (InvivoGen, USA). All cells were incubated in a humidified CO_2_ incubator at 37 °C.

### Generation of induced pluripotent stem cell-derived human blood vessel organoids

BVOs were generated and cultured following the protocol as previously described^21^. Briefly, hiPSC aggregates were generated by seeding in a low-attachment plate in StemFlex with 50 μM Y-27632. The next day, hiPSC aggregates were collected and the medium replaced with N2B27 medium supplemented with 12 μM CHIR99021 and 30 ng ml^−1^ BMP4. On day 3, the medium was replaced with N2B27 supplemented with 100 ng ml^−1^ VEGF-A (Peprotech) and 2 μM forskolin (STEMCELL Technologies). On day 5, aggregates with a diameter of 250–300 μm were selected and embedded in Collagen–Matrigel mixture (3:1 ratio) in 12-well Transwell inserts, with the aggregates placed in the centre of the extracellular matrix (ECM) solution. The cultures were maintained in StemPro-34 SFM medium (Gibco) supplemented with 15% fetal bovine serum (Gibco), 100 ng ml^−1^ VEGF-A, and 100 ng ml^−1^ FGF2.

### Fused skin-vascular assembloid cultures

At approximately 85 days, each cyst-like SkO was cut into eight evenly sized portions and placed onto the ECM, on which BVOs (day 12 of BVO generation) had been formed. The VSAs were maintained for 3–4 weeks in an incubator. The skin maturation medium was changed every 3 days. To further induce epidermal maturation, the VSAs were transferred to a dry-conditioned incubator at 37 °C for an additional 6 days, and the medium was replaced daily.

### Immunofluorescence assay

The cryo-sectioned organoids were fixed with 4% paraformaldehyde. After washing with PBS, the samples were blocked with 5% normal goat serum and incubated with the primary antibodies overnight at 4 °C (Supplementary Table 1). After PBS washing, fluorescence-conjugated secondary antibodies (Invitrogen, USA) were applied for 1 h at room temperature. For nuclei staining, 4,6-diamidino-2-phenylindole dihydrochloride (DAPI, Invitrogen) was applied for 10 min at room temperature. The stained samples were mounted with fluorescent mounting medium (DAKO, Denmark) and visualized using a confocal microscope (Eclipse TE2000, Nikon, Japan) with EZ-C1 3.8 software (Nikon) or a THUNDER Imaging System (Leica Microsystems; NFEC-2025-09-308232). The mean fluorescence intensity or the number of cells expressing the protein of interest was quantified by ImageJ software.

### Single-cell RNA sequencing and analysis

Single-cell transcriptome profiling of SkOs and VSAs generated from hiPSCs was performed. Organoids and assembloids were dissociated using the Whole Skin dissociation Kit, human (Miltenyi Biotec) for 4 h at 37 °C in a 1.5-ml microcentrifuge tube. Next, the organoids and assembloids were carefully washed with PBS, and residual tissue fragments were gently triturated to obtain single-cell suspensions. Single-cell libraries were constructed using Chromium Next Gem Single Cell 3′ Reagent Kits v4 (10x Genomics) and sequenced on an Illumina NovaSeq X Plus platform (paired-end 150 bp). FASTQ files were processed with Cell Ranger (v9.0, 10x Genomics) to generate gene-expression matrices. Downstream analyses were performed using the Seurat package (v5.0.0, R), Loupe Cell Browser (10x Genomics), and Winseurat (ebiogen, Republic of Korea). Low-quality cells were filtered using standard thresholds, and datasets from SkO and VSA samples were normalized, integrated and clustered. Dimensionality reduction was conducted with principal component analysis and uniform manifold approximation and projection. Cell-type annotation was guided by canonical lineage markers, and visualization was performed using Seurat plotting functions, ggplot2 and Loupe.

### Lucifer Yellow permeability assay

To assess epidermal barrier-associated permeability, Lucifer Yellow CH dipotassium salt (Invitrogen) was applied to the epidermal surface of SkOs and VSAs. After incubation for 4 h at 37 °C, samples were washed with PBS, fixed with 4% paraformaldehyde, embedded, cryosectioned and analysed by fluorescence microscopy. Lucifer Yellow penetration into the epidermal and dermal regions was quantified using ImageJ software.

### Histological analysis

For haematoxylin and eosin (H&E) staining, sections were stained with haematoxylin solution for 5 min, differentiated in 0.5% HCl in ethanol, and counterstained with eosin for 1 min. For Masson’s trichrome staining, sections were re-fixed in Bouin’s solution overnight at room temperature. The sections were then stained with Weigert’s haematoxylin for 10 min, followed by Biebrich scarlet acid fuchsin for 5 min. Differentiation was performed using a mixture of phosphotungstic acid, phosphomolybdic acid and distilled water (1:1:2) for 10 min. Subsequently, the slides were directly transferred to 2% aniline blue for 5 min and washed with 1% acetic acid. Following dehydration through a graded ethanol series, the samples were mounted with Canada Balsam. Stained sections were visualized with an Olympus microscope and ProgRes CapturePro software (Olympus, Japan).

### In vivo transplantation and vascular accessibility analysis

All animal experiments were performed in accordance with protocols approved by the Institutional Animal Care and Use Committee of Seoul National University (IACUC No. SNU-250310-3-1). 6-week-old female BALB/c-Foxn1nu/Arc mice were purchased from JA BIO Co., Ltd. and maintained under pathogen-free conditions at the Animal Center for Pharmaceutical Research of Seoul National University. To evaluate the in vivo persistence and vascular accessibility of VSA-derived vascular structures, VSAs were transplanted into mice. Briefly, mice were anaesthetized, and a full-thickness dorsal skin wound was generated using a 5-mm biopsy punch. VSAs were placed onto the wound bed and secured by suturing the surrounding host skin. Grafts were allowed to engraft for 4 weeks before analysis. To assess vascular accessibility, rhodamine-conjugated Ulex europaeus agglutinin I (UEA-1) was intravenously injected before tissue harvest. Graft-containing skin tissues were collected, fixed with 4% paraformaldehyde, embedded, sectioned and analysed by histological and immunofluorescence staining. Human nuclear antigen, human CD31, mouse CD31 and UEA-1 staining were used to assess graft-derived human tissue, host–graft vascular association and vascular accessibility.

### Umbilical cord blood-mononuclear cell isolation

Umbilical cord blood (UCB) was obtained from the Seoul metropolitan Government with the approval of the Seoul National University Institutional Review Board (IRB-No.E2405/002-021). Human mononuclear cells (MNCs) were isolated as described previously^22^. In brief, UCB samples from independent healthy donors were diluted in PBS at a 1:1 ratio, and human MNCs were isolated from the supernatants by Ficoll gradient centrifugation (Ficoll-Paque PLUS, GE Healthcare). MNCs from each donor were independently incorporated into VSAs to assess the reproducibility of immune responses.

### Co-culture of vascularized skin assembloids and mononuclear cells

For the establishment of co-culture systems, 12-well Transwell inserts (Corning) were pre-coated with 150 μl of collagen solution and allowed to gel at 37 °C. MNCs (3×10^5^ cells) were suspended in 500 μl of MNC medium and seeded onto the collagen gel within the inserts. After 24 h of incubation, the culture medium was removed, and the VSAs were subsequently placed onto the inserts. Co-cultures were maintained in a mixed medium composed of skin maturation medium and MNC medium at a 1:1 ratio. The MNC medium consisted of RPMI1640 (Gibco) supplemented with 10% FBS and Primocin.

### Immune stimulation with lipopolysaccharides

To evaluate the immune responsiveness of immune-supplemented VSAs, cultures were stimulated with lipopolysaccharides (LPS) (100 ng ml^−1^; Sigma-Aldrich). LPS stimulation was performed twice at 2-day intervals, and assembloids were harvested 2 days after the final treatment for downstream analyses and histological assessment.

### Allergen-induced inflammatory modelling

To establish an HDM-induced inflammatory model, VSAs were stimulated with HDM extract (*Dermatophagoides pteronyssinus*; Citeq, Netherlands) at a dose of 5 µg per sample (in 5 µl). HDM stimulation was performed three times at 2-day intervals, and samples were harvested 3 days after the final treatment for cytokine analysis, immunostaining and transcriptomic profiling.

### Flow cytometric analysis of immune cells in vascularized skin assembloid–mononuclear cell co-cultures

To characterize immune-cell populations incorporated into VSAs, VSA–MNC co-cultures were dissociated into single-cell suspensions at the indicated time points using the Whole Skin Dissociation Kit, human (Miltenyi Biotec). The cell suspensions were filtered, washed with FACS buffer and stained with the StainExpress Immune Cell Composition Cocktail, human (Miltenyi Biotec), or with fluorophore-conjugated antibodies against CD45, CD3, CD11c, HLA-DR and CD1c for myeloid/APC-like cell analysis. Dead cells were excluded using a viability dye. Samples were analysed using a MACSQuant Analyzer 10 flow cytometer (Miltenyi Biotec). Compensation was performed using MACS Comp Beads and the MultiColor Compensation function. Flow cytometry data were analysed using MACSQuantify software.

### Quantitative real-time PCR

One sample of SkOs from each group was washed with PBS and lysed in TRIzol (Macherey-Nagel, Germany) for total RNA extraction. Complementary DNA synthesis was performed using a Superscript III First-Strand synthesis system kit (Invitrogen) according to the manufacturer’s instructions. Quantitative real-time PCR analysis was performed with SYBR Green PCR Mix (Applied Biosystems, USA) on a 7500 Real-Time PCR system (Applied Biosystems). The relative mRNA expression of each target gene was quantified by the 2-ΔΔCt method and normalized to the housekeeping gene, glyceraldehyde-3-phosphate dehydrogenase (GAPDH) (Supplementary Table 2).

### RNA sequencing and bioinformatics analysis

Total RNA was extracted from organoids using TRIzol reagent and libraries were prepared with a commercial RNA-sequencing (RNA-seq) library preparation kit. Sequencing was performed on the Illumina NextSeq 2000 platform with paired-end reads. Raw data were aligned to the human reference genome (GRCh38), and differentially expressed genes were identified based on fold-change and statistical thresholds. Heatmaps and volcano plots were generated to visualize gene-expression patterns. Gene ontology and Kyoto Encyclopedia of Genes and Genomes pathway enrichment analyses were performed, and gene set enrichment analysis was conducted to evaluate functional pathways. To benchmark the HDM-induced VSA transcriptomic response against human atopic dermatitis (AD), a publicly available bulk RNA-seq dataset of skin biopsies from healthy controls and patients with AD was used (GSE277961).

### Enzyme-linked immunosorbent assay

To analyse the secretion of VSAs under both baseline and HDM-exposed conditions, the supernatant of conditioned media was collected and subjected to enzyme-linked immunosorbent assay (ELISA). To assess endothelial-specific functions, the concentrations of human VEGF and nitric oxide were quantified using the Human VEGF Quantikine ELISA Kit (DVE00, R&D Systems) and the Nitric Oxide Detection Kit (iNtRON Biotechnology, Republic of Korea), respectively. To evaluate immune-related responses in the HDM-induced inflammatory model, the levels of thymic stromal lymphopoietin (TSLP; DY1398, R&D Systems), TARC/CCL17 (DY364, R&D Systems), IL-4 (DY204, R&D Systems) and IL-13 (DY213, R&D Systems) were measured using the corresponding human ELISA kits according to the manufacturer’s instructions.

### Western blot

Sample lysates were extracted by homogenization with PRO-PREP (iNtRON Biotechnology), followed by sonication. The 20 μg of proteins were separated by sodium dodecyl sulfate-polyacrylamide gel electrophoresis and transferred to polyvinylidene difluoride membranes. The membranes were blocked with 3% bovine serum albumin at room temperature for 1 h and then incubated on an agitator with the primary antibodies at 4 °C overnight (Supplementary Table 3). Membranes were washed and incubated with secondary antibodies (Invitrogen) at room temperature for 1 h. Each band was detected with an enhanced chemiluminescence detection kit (Cytiva, USA).

### Proteome profiler arrays

Conditioned media collected from VSAs were subjected to cytokine profiling using the Proteome Profiler Human XL Cytokine Array Kit (R&D Systems) in accordance with the manufacturer’s protocol. Membranes were incubated with the samples, and bound cytokines were detected by chemiluminescence. Signal intensities were quantified using ImageJ software, and relative levels were compared across experimental groups.

### Statistical analysis

Sample size (*n*) represents the number of biologically independent replicates, with at least three independent experiments performed for each condition. All values are presented as mean ± standard deviation (SD), and all graphs were generated using GraphPad Prism version 10.0. Statistical analyses were performed using unpaired two-tailed *t* tests, one-way analysis of variance with Tukey’s post-hoc test, or two-way analysis of variance with Bonferroni’s post-hoc test. Statistical significance was denoted as *P < 0.05, **P < 0.01 and ***P < 0.001. For single-cell analyses, proportions of marker-positive cells were compared using Fisher’s exact test with Benjamini–Hochberg correction for multiple testing.

## Results

### Modular assembly of vascularized skin assembloids

**Fig. 1:**
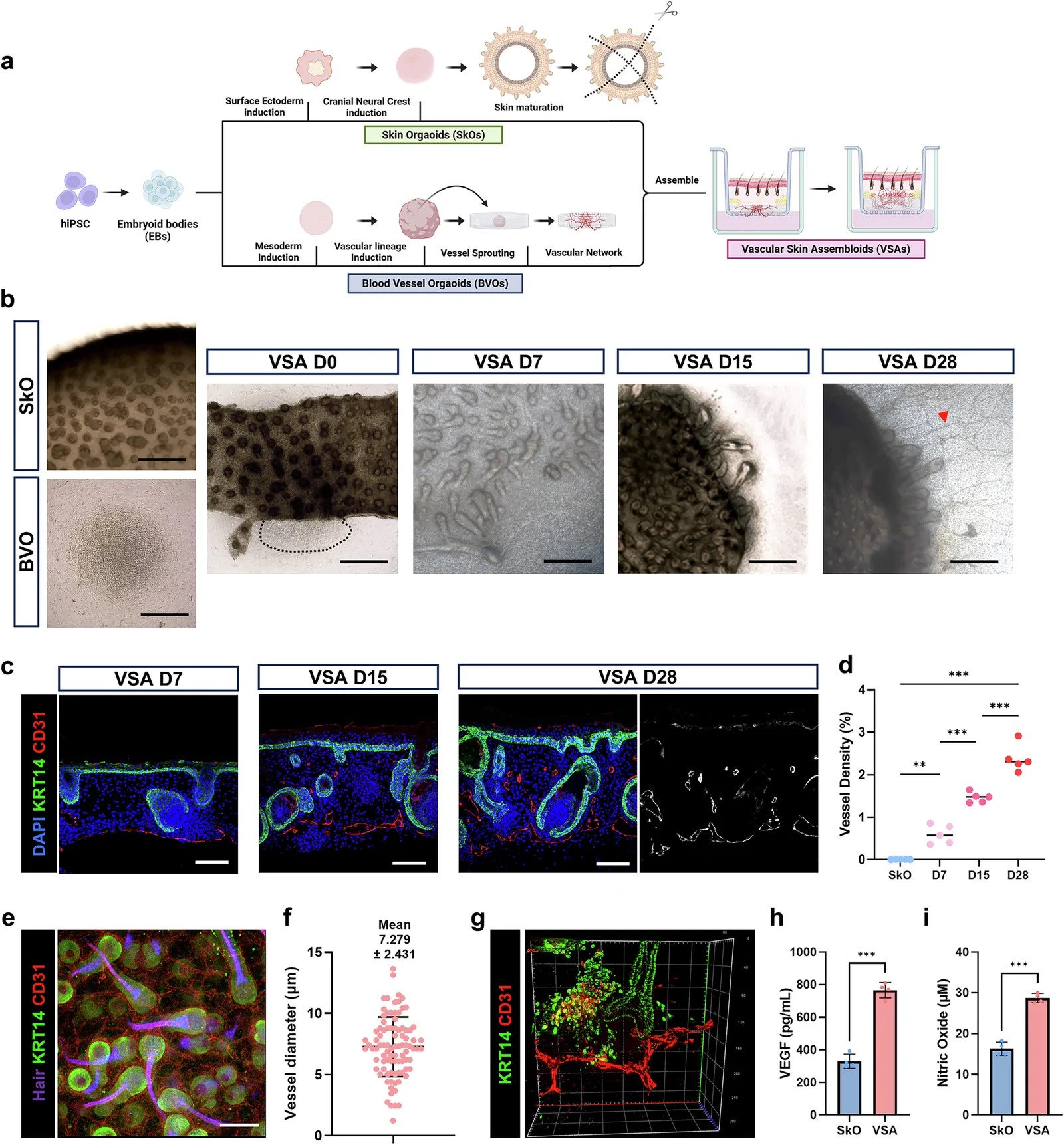
Generation of vascularized skin assembloids by modular integration of skin organoids and blood vessel organoids. **a** Schematic of the stepwise assembly of vascularized skin assembloids (VSAs) by combining human induced pluripotent stem cell (hiPSC)-derived skin organoids (SkOs) and blood vessel organoids (BVOs) under air–liquid interface culture. **b** Bright-field images showing individual SkO and BVO controls (left) and representative time course of VSA development (D0, D7, D15, D28). SkOs were positioned on pre-vascularized extracellular matrix at D0, expanded by D7, stabilized in thickness by D15 and exhibited extensive vascular branching by D28 (arrowheads). **c** Representative confocal images of VSAs stained with KRT14 (green), CD31 (red) and DAPI (blue). **d** Quantification of the vascularized area during VSA maturation (*n* = 5). **e** Horizontal z-stack confocal projections showing three-dimensional CD31^+^ vascular networks (red) surrounding KRT14^+^ epidermal/follicular compartments (green). **f** Distribution of vessel diameter within VSAs (*n* = 30). **g** Vertical cryosection and 3D reconstruction illustrating spatial alignment of epidermal and vascular domains. **h, i** Quantification of VEGF (**h**) and nitric oxide (**i**) secretion in SkOs and VSAs (*n* = 3). All scale bars, 100 μm. Data are presented as mean ± standard deviation. Statistical differences were determined by unpaired Student’s *t* test or one-way analysis of variance with Tukey’s post hoc test (*P < 0.05, **P < 0.01, ***P < 0.001). Figure 1a was created with BioRender.com.

To overcome the limitations of conventional SkOs, which lack vasculature and display architectural instability^13,14,16^, VSAs were established by modularly integrating hiPSC-derived SkOs with BVOs under air–liquid interface conditions (Fig. 1a). SkOs exhibited cyst-like morphology with evidence of appendage formation, including early hair-follicle structures (Supplementary Fig. 1a). In parallel, BVOs formed endothelial networks with emerging luminal structures when embedded in collagen–Matrigel composite ECM (Supplementary Fig. 1b, c). Notably, vascular density and stability were strongly influenced by ECM composition^23^. *PECAM1* expression, reflecting endothelial density, was highest under a Matrigel:collagen (M:C) 1:3 condition. *CDH5*, a marker of endothelial junctional organization, was also maximally expressed under this condition. In contrast, *α-SMA* expression progressively increased with higher collagen ratios and peaked in collagen-only matrices, which may reflect mural-cell activation in collagen-rich environments, potentially affecting vascular organization. Taken together, these analyses identified the (M:C) 1:3 ratio as the optimal ECM composition for VSA assembly, balancing vascular density with junctional organization while minimizing aberrant α-SMA induction (Supplementary Fig. 1d).

Using this optimized ECM condition, the temporal development of VSAs was achieved by integrating SkOs with BVOs. At D0, SkOs and BVOs were physically juxtaposed within the matrix but retained distinct boundaries. At D7, SkOs fused with the pre-vascularized ECM and spread along its surface, initiating structural integration. At D15, the fused structure stabilized and increased in thickness, consistent with early tissue consolidation. By D28, vascular-like extensions infiltrated the ECM and progressively elaborated into branched networks projecting from the dermal compartment (Fig. 1b). Gene-expression analysis corroborated these morphological observations, showing that *PECAM1* and *CDH5* levels increased gradually over time, reflecting endothelial network development^24^. In contrast, *α-SMA* rose only moderately, consistent with organized vascular-like network formation. In parallel, *KRT10* expression progressively increased, consistent with epidermal differentiation and stabilization, whereas barrier-associated genes *FLG* and *LOR* were significantly upregulated by D28, supporting progressive barrier formation (Supplementary Fig. 1e). Immunofluorescence revealed progressive vascular organization within the dermal compartment. At D7, BVO-derived vascular structures localized beneath the dermis, whereas at D15, CD31^+^ branches sprouted upwards. By D28, CD31^+^ vascular-like networks arborized extensively throughout the dermis, forming dense interconnected structures (Fig. 1c, d). Three-dimensional reconstructions demonstrated that CD31^+^ networks maintained relatively uniform vessel diameters and organized structures within the dermal compartment (Fig. 1e, f). Notably, follicular structures were often localized adjacent to CD31^+^ vessels, indicating spatial association between skin appendages and vasculature (Fig. 1g). Functionally, VSAs exhibited elevated nitric oxide production and increased VEGF secretion compared with non-vascularized controls, indicating endothelial-associated activity (Fig. 1h, i). Collectively, these results demonstrate that modular integration of hiPSC-derived SkOs with BVOs under optimized ECM conditions leads to the formation of organized interconnected vascular-like networks within the dermal compartment of VSAs. This assembly approach supports coordinated epidermal differentiation, dermal–vascular organization and endothelial activity, thereby providing a foundation for constructing human-relevant VSA models.

### Single-cell transcriptomic profiling supports multicellular complexity and vascular-associated populations in vascularized skin assembloids

**Fig. 2:**
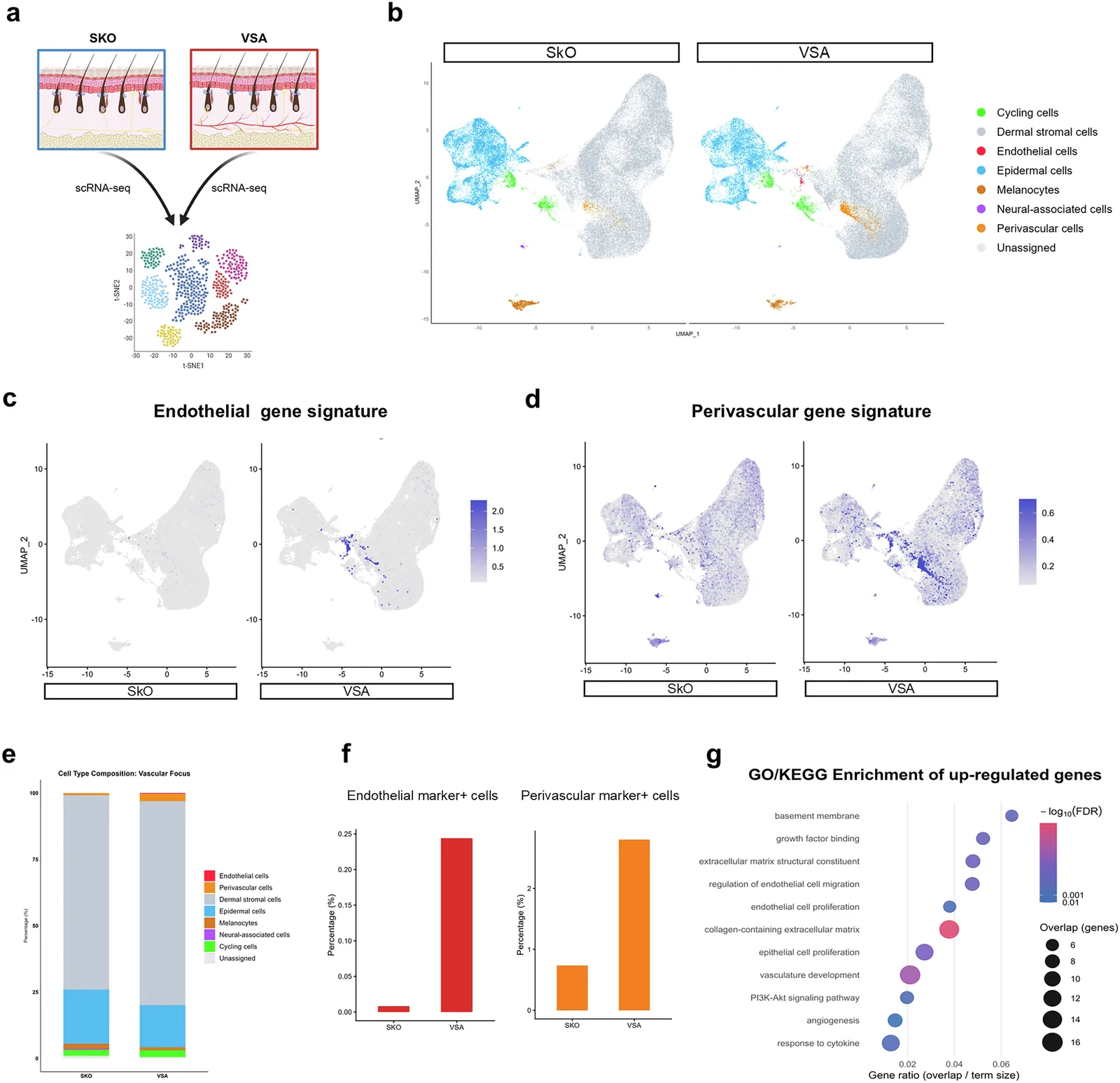
Single-cell transcriptomic profiling reveals multicellular organization and vascular enrichment in vascularized skin assembloids. **a** Schematic overview of the single-cell RNA sequencing (scRNA-seq) workflow comparing skin organoid (SkOs) and vascularized skin assembloids (VSAs). **b** Uniform manifold approximation and projection (UMAP) visualization of integrated scRNA-seq datasets split by SkO and VSA, with annotation of major skin-associated populations, including epidermal cells, dermal stromal cells, cycling cells, melanocytes, neural-associated cells, endothelial cells, perivascular cells and unassigned cells. **c** UMAP feature plots showing endothelial gene-signature scores based on canonical endothelial markers, including *PECAM1*, *CDH5*, *KDR*, *VWF* and *CLDN5*, in SkOs and VSAs. **d** UMAP feature plots showing perivascular gene signature scores based on perivascular/mural-associated markers, including *PDGFRB*, *RGS5*, *MCAM*, *ACTA2*, *DES*, *KCNJ8* and *ABCC9*, in SkOs and VSAs. **e** Cell-type composition analysis of SkOs and VSAs based on marker-defined annotation, highlighting endothelial and perivascular populations. **f** Focused quantification of endothelial marker-positive and perivascular marker-positive cells as percentages of total captured cells in SkOs and VSAs. **g** Gene Ontology (GO)/Kyoto Encyclopedia of Genes and Genomes (KEGG) enrichment analysis of genes upregulated in VSAs relative to SkOs, showing vascular-, extracellular matrix-, and cytokine response-associated biological programs. scRNA-seq was performed using two biological replicates per group (SkO, *n* = 2; VSA, *n* = 2). Statistical significance was determined by Fisher’s exact test with Benjamini–Hochberg correction (*False Discovery Rate (FDR) < 0.05, **FDR < 0.01, ***FDR < 0.001). Figure 2a was created with BioRender.com.

To systematically compare the cellular composition of VSAs and SkOs, single-cell RNA sequencing was performed followed by integrated clustering analysis. Uniform manifold approximation and projection revealed shared and distinct cellular populations between SkOs and VSAs, which were annotated into major skin-associated lineages, including epidermal cells, dermal stromal cells, cycling cells, melanocytes, neural-associated cells, endothelial cells and perivascular cells (Fig. 2a, b). Representative marker analysis supported these annotations, with epidermal, dermal stromal, cycling, melanocyte, neural-associated, endothelial and perivascular markers showing distinct expression patterns across the annotated populations (Supplementary Fig. 2a–c).

We next examined vascular-associated populations using marker-defined gene signatures rather than broad cluster-level annotation alone. Endothelial-associated signatures, based on canonical markers including *PECAM1*, *CDH5*, *KDR*, *VWF* and *CLDN5*, were more evident in VSAs than in SkOs (Fig. 2c). Perivascular-associated signatures, including *PDGFRB*, *RGS5*, *MCAM*, *ACTA2*, *DES*, *KCNJ8* and *ABCC9*, were also increased in VSAs compared with SkOs (Fig. 2d). Consistent with these signature analyses, marker-defined cell composition showed increased endothelial and perivascular populations in VSAs compared with SkOs (Fig. 2e). Focused quantification further confirmed higher proportions of endothelial marker-positive and perivascular marker-positive cells following BVO integration (Fig. 2f). Additional marker-level analyses in the supplementary figures further supported increased endothelial and perivascular marker expression in VSAs (Supplementary Fig. 2d,e).

To assess biological programmes associated with VSA formation, enrichment analysis of VSA-upregulated genes was performed. This analysis revealed vascular- and ECM-related biological terms, including endothelial-cell proliferation, regulation of endothelial-cell migration, vasculature development, angiogenesis, basement membrane organization, ECM structural constituent, PI3K–Akt signalling, and cytokine response pathways (Fig. 2g). Together, these single-cell transcriptomic analyses show that VSAs retain diverse skin-associated lineages and exhibit increased endothelial and perivascular marker-defined populations, consistent with the vascular structures observed by histological and immunofluorescence analyses.

### Characterization of structural and cellular features of vascularized skin assembloids

**Fig. 3:**
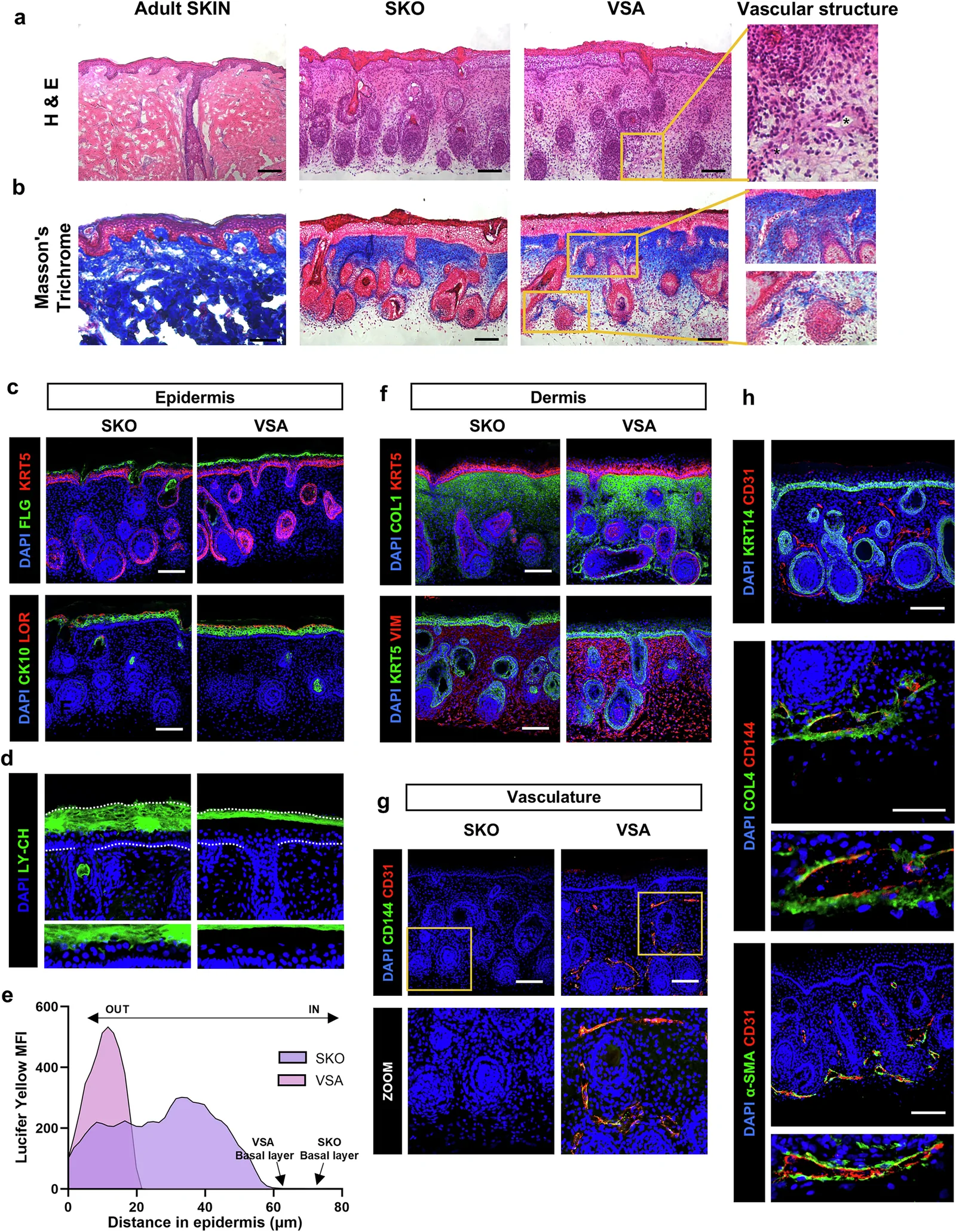
Histological and immunophenotypic characterization of skin organoids and vascularized skin assembloids relative to adult skin. **a** Representative haematoxylin and eosin (H&E) staining images of adult skin, skin organoids (SkOs) and vascularized skin assembloids (VSAs) showing stratified epidermis and underlying dermal compartments. VSAs exhibited expanded dermal regions with lumenized vascular-like structures (yellow boxes, magnified insets). **b** Masson’s trichrome staining demonstrates dense collagen deposition (blue) surrounding vascular regions in VSAs. **c** Immunofluorescence images of epidermal organization with filaggrin (FLG) (green), KRT5 (red) and DAPI (blue); KRT10 (green), LOR (red) and DAPI (blue). **d** Lucifer Yellow permeability assay showing dye penetration from the epidermal surface into SkOs and VSAs. Dotted lines indicate the epidermal region. **e** Quantification of Lucifer Yellow fluorescence intensity across the epidermal depth in SkOs and VSAs. **f** Confocal images illustrating dermal architecture with COL1A1^+^ extracellular matrix (green) and VIM^+^ fibroblasts (red) located beneath the KRT5^+^ basal epidermal layer. **g**, **h** Immunostaining for vascular endothelial markers CD31 and CD144 (red/green) and perivascular markers α-SMA, COL4 and CD144 showing organized vessel-like networks associated with the basement membrane and perivascular compartment. Insets highlight lumen-like vascular structures. All scale bars, 100 μm. MFI, mean fluorescence intensity.

Previous studies have shown that skin organoids can generate stratified epidermal and dermal compartments, but they generally fail to establish vascular structures, a defining feature of native human skin^13,16^. To address this limitation, we comprehensively compared the histological and immunophenotypic features of SkOs and VSAs relative to adult skin. Histological analysis revealed that both models developed a stratified epidermis above a fibroblast-rich dermis; however, VSAs uniquely exhibited an expanded dermal region containing lumenized vascular structures, showing structural features reminiscent of adult skin (Fig. 3a). Masson’s trichrome staining further demonstrated dense collagen deposition surrounding vascular regions, suggesting spatial association between vasculature and the ECM (Fig. 3b). Immunofluorescence showed that VSAs developed an organized epidermis with a defined KRT5^+^ basal layer and KRT10^+^ suprabasal layer. Barrier-associated proteins filaggrin (FLG) and loricrin (LOR), important markers of epidermal differentiation and barrier formation^25^, were more prominently expressed in VSAs than in SkOs (Fig. 3c; Supplementary Fig. 3a, b). To further assess barrier-associated functionality, we performed a Lucifer Yellow permeability assay. Compared with SkOs, VSAs showed reduced Lucifer Yellow penetration into deeper epidermal regions, with fluorescence largely restricted to the outer epidermal layer (Fig. 3d, e). These results indicate that VSAs develop a more mature barrier-associated epidermal phenotype and improved restriction of dye penetration compared with conventional SkOs.

Within the dermis, Vimentin^+^ fibroblasts and Collagen I^+^ ECM were arranged directly beneath the epidermis, supporting dermal–epidermal compartmental organization, a structural feature relevant to human skin organization^26^ (Fig. 3f, Supplementary Fig. 3c). Vascular compartmentalization was further supported by endothelial junctional markers. CD31 and VE-cadherin (CD144) formed continuous, network-like structures resembling vessel junctions in VSAs, whereas SkOs lacked such organization (Fig. 3g). Importantly, additional analyses revealed features of vascular organization: CD31^+^ endothelial networks were positioned beneath the KRT14^+^ basal epidermis, ensheathed by α-SMA^+^ perivascular cells and surrounded by Collagen IV deposition aligned with VE-cadherin^+^ junctions, together forming vessel-like tubes associated with basement-membrane components (Fig. 3h). To further characterize vascular identity and accessibility, additional analyses were performed. CD31^+^ endothelial structures in VSAs showed expression of artery- and vein-associated markers, including SOX17 and COUP-TFII, respectively (Supplementary Fig. 3d). In contrast, definitive PROX1^+^ lymphatic vessel-like structures were not clearly observed under the current culture conditions (Supplementary Fig. 3e).

Finally, in vivo transplantation analysis showed that VSA grafts persisted in BALB/c nude mice after 4 weeks and contained human nuclear antigen-positive graft-derived tissue (Supplementary Fig. 3f, g). Human CD31^+^ vascular structures were detected within the grafted region and were closely associated with mouse CD31^+^ host vasculature (Supplementary Fig. 3h). Intravenous injection of rhodamine-conjugated UEA-1 further revealed UEA-1-accessible human CD31^+^ vascular structures within transplanted VSAs, supporting the in vivo vascular accessibility of VSA-derived vascular structures (Supplementary Fig. 3i). In summary, histological, molecular and functional characterization supported the presence of key skin structural features, including a stratified epidermis, enhanced epidermal barrier restriction, organized dermal matrix and vascular-like networks associated with basement membrane and perivascular components. Collectively, these findings demonstrate that VSAs display enhanced structural and vascular organization compared with conventional SkOs, providing a more structurally complex vascularized skin assembloid model.

### Integration of mononuclear cells into vascularized skin assembloids provides an immune-responsive skin model

**Fig. 4:**
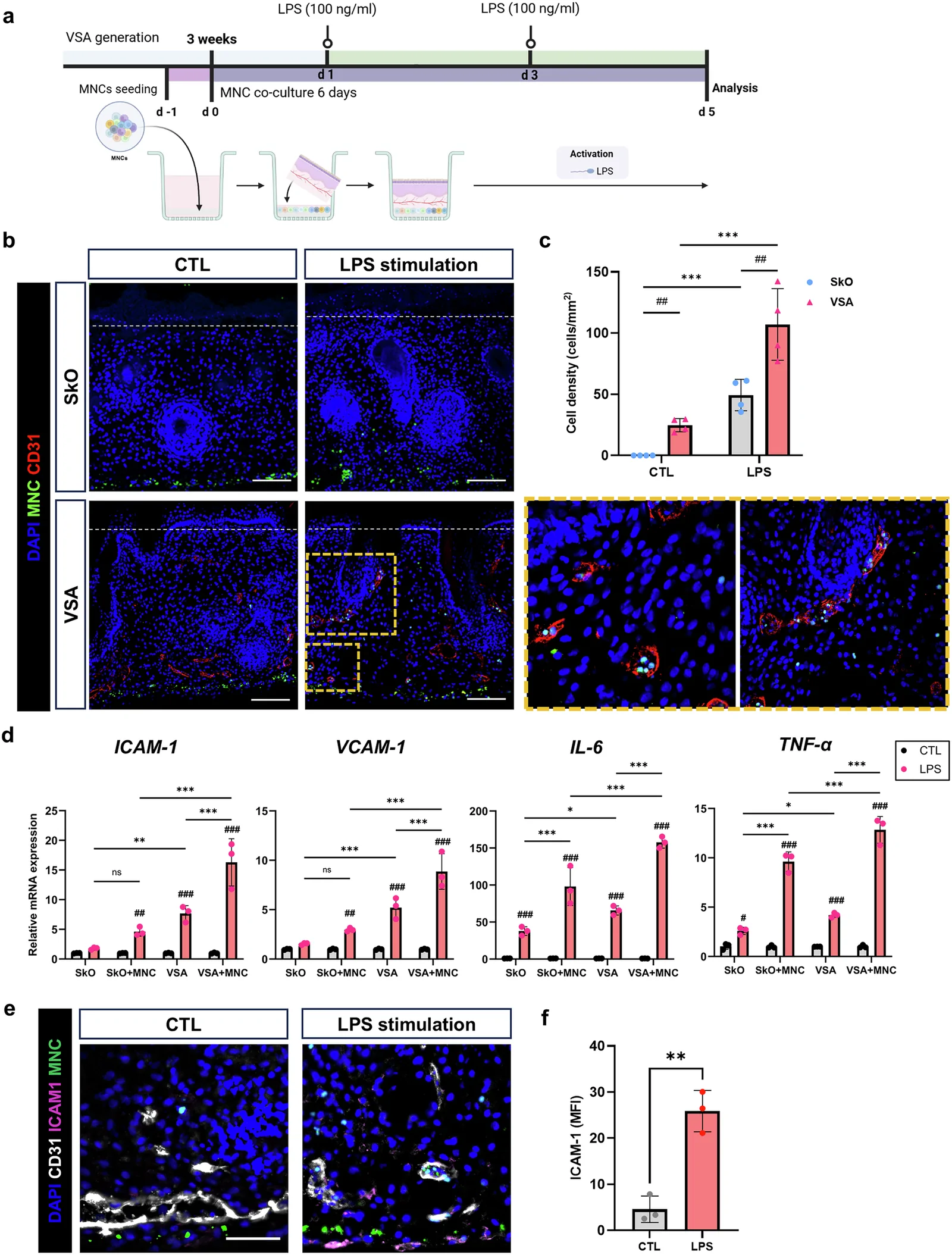
Immune-integrated vascularized skin assembloids reveal vascular-associated immune-cell infiltration and activation. **a** Schematic of experimental workflow for generating immune-integrated vascularized skin assembloids (VSAs) by integrating umbilical cord blood-derived mononuclear cells (MNCs) followed by lipopolysaccharide (LPS) stimulation. **b** Representative immunofluorescence images showing MNCs (green) accumulated near CD31^+^ vascular structures (red) within the upper dermis, in contrast to avascular skin organoids (SkOs). Insets highlight perivascular localization. **c** Quantification of MNC density within the upper dermis (0–300 μm above the dermal–epidermal junction) (*n* = 4). **d** Quantitative PCR analysis of adhesion molecules (ICAM1, VCAM1) and cytokines (IL-6, TNFα) across four conditions (SkO, SkO+MNC, VSA, VSA + MNC). **e,** **f** Representative immunofluorescence images and quantification showing increased ICAM1 expression in LPS-stimulated VSAs compared with controls. ICAM1 signals were detected in association with CD31^+^ vascular-like structures. DAPI, blue; CD31, white; ICAM1, magenta; MNCs, green. All scale bars, 100 μm. Data are presented as mean ± standard deviation. Statistical differences were determined by two-way analysis of variance with Bonferroni’s post hoc test or unpaired Student’s *t* test (*P < 0.05, **P < 0.01, ***P < 0.001; #*P* < 0.05, ##*P* < 0.01, ###*P* < 0.001). Figure 4a was created with BioRender.com. MFI, mean fluorescence intensity; mRNA, messenger RNA.

We developed immune-integrated VSAs by integrating umbilical cord blood-derived MNCs to investigate whether vascularization influences immune cell behaviour. Following assembly, green-labelled MNCs were seeded onto the collagen matrix and co-cultured with VSAs for 24 h, followed by LPS stimulation (Fig. 4a). Upon LPS stimulation, MNCs accumulated within the upper dermal region of VSAs and were frequently observed in close proximity to CD31^+^ vascular structures, whereas in matched non-vascularized SkOs, infiltrating cells rarely progressed beyond the lower dermis (Fig. 4b). Quantitative analysis of MNC density within a defined upper-dermis window (0–300 μm above the dermal–epidermal junction, DEJ) revealed a significant increase in VSAs compared with SkOs (Fig. 4c). To assess inflammatory activation at the transcriptional level, we performed quantitative PCR for adhesion molecules (*ICAM1*, *VCAM1*) and pro-inflammatory cytokines (*IL-6*, *TNFA*) across four conditions (SkO, SkO+MNCs, VSA, VSA+MNCs). Following LPS stimulation, avascular SkOs showed only modest induction, whereas vascularized constructs exhibited markedly higher responses, with the strongest activation observed in VSA+MNCs. Under identical stimulation, VSA+MNCs expressed significantly higher levels of all four genes than SkO+MNCs, suggesting that vascularized VSAs support enhanced innate inflammatory signalling (Fig. 4d). Consistent with these findings, immunofluorescence analysis confirmed the presence of CD31^+^ vascular structures in both groups and demonstrated robust ICAM1 upregulation in LPS-treated VSAs, localized primarily to CD31^+^ vascular structures, with infiltrating MNCs situated in close proximity to ICAM1^+^ endothelial segments (Fig. 4e, f). Collectively, these results indicate that integration of MNCs into VSAs supports immune-cell infiltration and endothelial inflammatory activation upon inflammatory challenge, providing an immune-responsive human skin model for investigating vascular–immune interactions.

### House dust mite stimulation induces atopic dermatitis-relevant inflammatory transcriptional programmes in vascularized skin assembloids

**Fig. 5:**
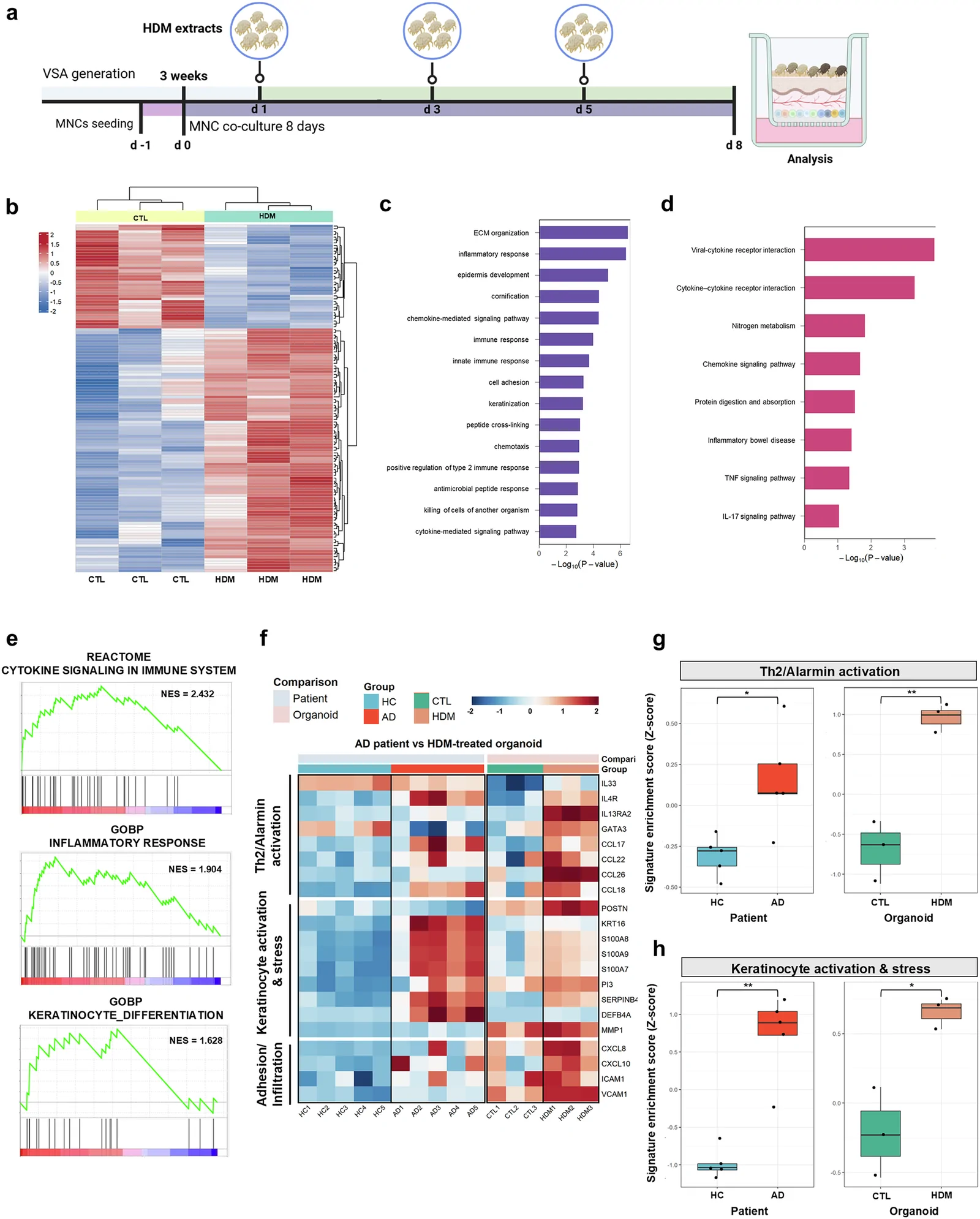
Transcriptomic analysis of house dust mite-treated vascularized skin assembloids reveals atopic dermatitis-relevant inflammatory signatures. **a** Schematic illustration of house dust mite (HDM) treatment regimen. **b** Hierarchical clustering heatmap showing distinct transcriptional separation between control and HDM-treated vascularized skin assembloids (VSAs). **c**, **d** Gene Ontology (**c**) and Kyoto Encyclopedia of Genes and Genomes (**d**) enrichment analyses of genes upregulated in HDM-treated VSAs, showing enrichment of extracellular matrix organization, inflammatory response, epidermal development, cornification, chemokine-mediated signalling, immune response, cytokine–cytokine receptor interaction, chemokine signalling, TNF signalling and IL-17 signalling pathways. **e** Gene set enrichment analysis plots showing enrichment of inflammatory and keratinocyte-associated pathways. **f** Z-score-normalized heatmap comparing atopic dermatitis (AD)-associated gene modules between published skin samples from healthy controls (HC) and patients with AD and control (CTL) and HDM-treated VSAs. Gene modules include type 2/alarmin activation, keratinocyte activation and stress, and adhesion/infiltration-associated mediators. **g**, **h** Signature enrichment score analysis comparing type 2/alarmin activation (**g**) and keratinocyte activation/stress (**h**) signatures in skin from HCs versus patients with AD and CTL versus HDM-treated VSAs. Bulk RNA-sequencing was performed using three biological replicates per group (CTL, *n* = 3; HDM, *n* = 3). Patient dataset comparison included skin samples from HCs and patients with AD as indicated in the heatmap. Data are presented as mean ± standard deviation. Statistical differences were determined by unpaired Student’s *t* test (*P < 0.05, **P < 0.01, ***P < 0.001). Figure 5a was created with BioRender.com. GOBP, Gene Ontology biological process; NES, normalized enrichment score; MNC, mononuclear cell.

These immune-responsive VSAs were used to model HDM-induced inflammatory remodelling with relevance to AD, using HDM extract, a major environmental allergen^27,28^. To induce this HDM-mediated inflammatory condition, immune-integrated VSAs were exposed to HDM extract (5 μg every 2 days, three treatments) (Fig. 5a). Fluorescence imaging confirmed allergen penetration into the dermal compartment, directly exposing dermal and vascular structures (Supplementary Fig. 4a, b). Transcriptomic profiling revealed extensive reprogramming in HDM-treated VSAs, with hierarchical clustering clearly separating control and HDM groups, indicative of a reproducible transcriptional divergence (Fig. 5b). Gene ontology analysis highlighted biological processes related to inflammatory response, cornification, immune activation and type 2 immune regulation, whereas Kyoto Encyclopedia of Genes and Genomes enrichment identified cytokine–cytokine receptor interaction, TNF signalling and IL-17 signalling cascades, pathways implicated in AD-associated inflammation (Fig. 5c, d). Gene Set Enrichment Analysis further corroborated these findings, showing enrichment of immune- and inflammation-related pathways, including cytokine signaling in immune system and inflammatory response, together with keratinocyte-associated pathways such as keratinocyte differentiation (Fig. 5e). To further evaluate the disease relevance of the HDM-induced transcriptional response, we compared the VSA transcriptomic profile with published transcriptomic data from skin from healthy controls and patients with AD^29^. A z-score-normalized heatmap showed that HDM-treated VSAs displayed AD-associated gene-expression patterns that partially paralleled those observed in skin from patients with AD, including type 2/alarmin-associated genes (*IL33*, *IL4R*, *IL13RA2*, *CCL17*, *CCL26* and *GATA3*), keratinocyte activation and stress-response genes (*POSTN*, *KRT16*, *S100A8*, *S100A9*, *PI3* and *SERPINB4*), and adhesion/infiltration-associated mediators (*CXCL8*, *CXCL10*, *ICAM1* and *VCAM1*) (Fig. 5f). Signature score analysis further showed increased type 2/alarmin activation and keratinocyte activation/stress signatures in both AD patient skin and HDM-treated VSAs compared with their respective controls (Fig. 5g, h). Adhesion/infiltration-associated signatures also showed a similar increasing trend in both datasets (Supplementary Fig. 4c). These transcriptional features indicate that HDM-treated VSAs exhibit selected AD-associated molecular features, particularly type 2/alarmin activation, keratinocyte stress responses and adhesion/infiltration-related inflammatory programmes.

### House dust mite stimulation induces barrier perturbation and immune activation in vascularized skin assembloids

**Fig. 6:**
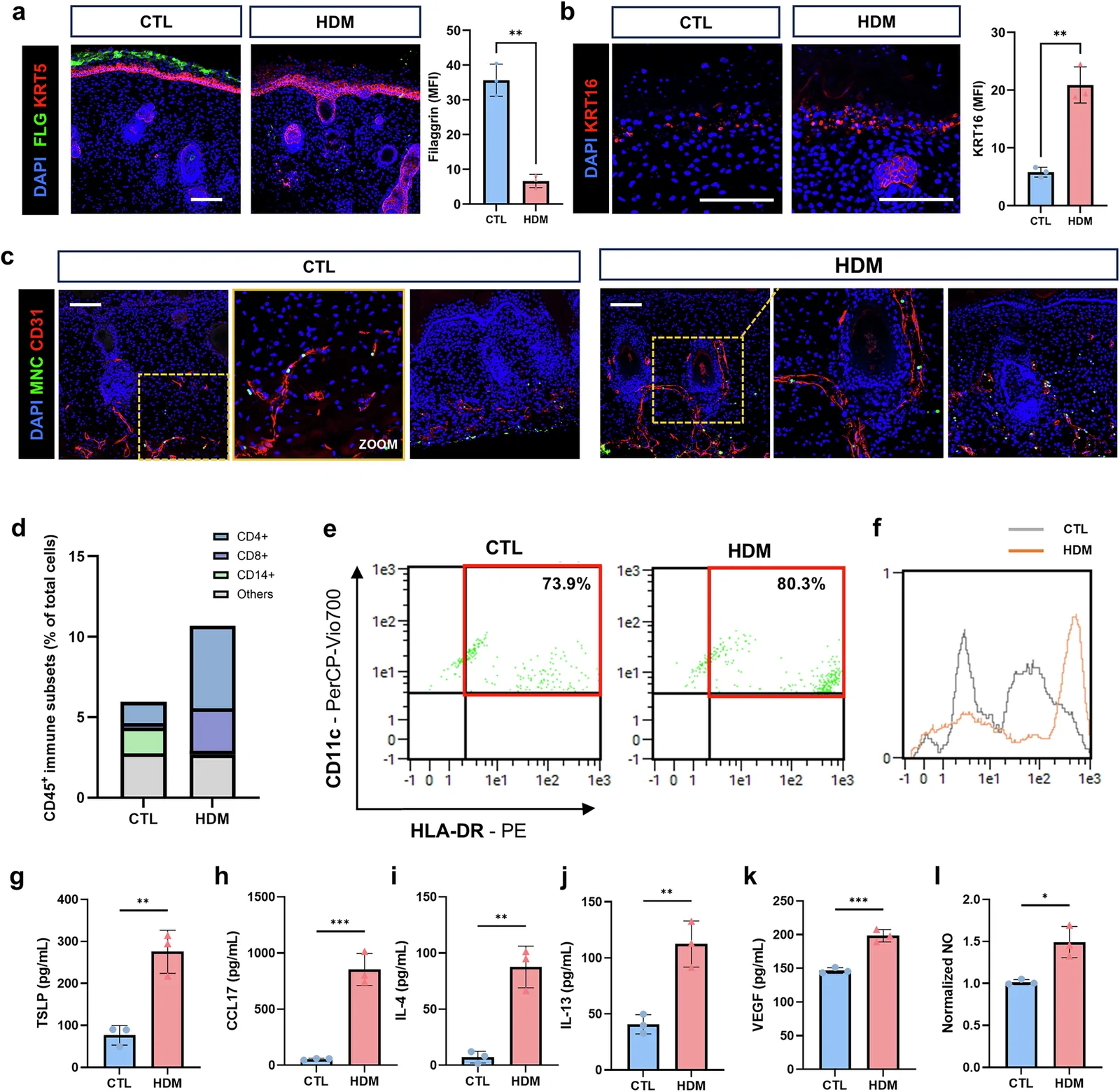
Barrier-associated and immune responses of vascularized skin assembloids following house dust mite stimulation. **a** Representative immunofluorescence images and quantification showing reduced filaggrin (FLG) expression in house dust mite (HDM)-treated vascularized skin assembloids (VSAs) compared with controls (CTL). DAPI, blue; FLG, green; KRT5, red. **b** Representative immunofluorescence images and quantification showing increased KRT16 expression in HDM-treated VSAs compared with controls. DAPI, blue; KRT16, red. **c** Representative immunofluorescence images showing mononuclear cell (MNC) infiltration and localization near CD31^+^ vascular-like structures in CTL and HDM-treated VSAs. DAPI, blue; MNCs, green; CD31, red. Yellow boxes indicate magnified regions. **d** Flow cytometric analysis of CD45^+^ immune-cell subsets recovered from VSA–MNC co-cultures, including CD4^+^ T cells, CD8^+^ T cells, CD14^+^ myeloid-lineage cells, and other CD45^+^ cells. **e** Representative flow cytometry plots showing CD11c^+^HLA-DR^+^ populations within the CD45^+^CD3^−^ immune-cell compartment in CTLs and HDM-treated VSAs. **f** Histogram showing HLA-DR signal intensity within CD11c^+^ myeloid/APC-like populations in CTLs and HDM-treated VSAs. **g–l** Enzyme-linked immunosorbent assay quantification of atopic dermatitis (AD)-associated inflammatory mediators and vascular-associated mediators in control and HDM-treated VSAs, including TSLP (**g**), CCL17/TARC (**h**), IL-4 (**i**), IL-13 (**j**), VEGF (**k**), and nitric oxide (NO; **l**). Data are presented as mean ± standard deviation. Statistical differences were determined by unpaired Student’s *t* test (*P < 0.05, **P < 0.01, ***P < 0.001). Scale bars, 100 μm. MFI, mean fluorescence intensity.

Histological analyses further supported these findings. H&E staining showed epidermal thickening in HDM-treated VSAs compared with controls, consistent with hyperplasia-like epidermal remodelling (Supplementary Fig. 5a, b). Consistently, immunofluorescence revealed reduced FLG and elevated KRT16 expression in the epidermis of HDM-treated VSAs, features associated with barrier perturbation and keratinocyte activation (Fig. 6a, b). Immune cells were largely absent from the upper dermis in controls, whereas in HDM-treated VSAs they were observed along vascular structures within the upper dermal regions (Fig. 6c, Supplementary Fig. 5c,d).

To further define the immune populations incorporated into VSAs, we performed flow cytometric analysis of VSA–MNC co-cultures. CD45^+^ immune cells recovered from VSAs contained representative immune subsets, including CD4^+^ T cells, CD8^+^ T cells and CD14^+^ myeloid-lineage cells, with an increased overall immune-cell fraction observed after HDM stimulation (Fig. 6d; Supplementary Fig. 5e). Because the current VSA platform does not contain bona fide epidermal Langerhans cells, we next examined whether MNC-derived APC-like populations were present within the incorporated immune compartment. Within the CD45^+^CD3^−^ population, CD11c^+^HLA-DR^+^ cells were detected in both control and HDM-treated VSAs and were modestly increased following HDM stimulation (Fig. 6e; Supplementary Fig. 5f). In addition, CD11c^+^CD1c^+^ populations were identified within the CD45^+^CD3^−^ compartment, supporting the presence of myeloid/APC-like immune populations in the VSA–MNC co-culture system (Supplementary Fig. 5g). Notably, HDM-treated VSAs showed a rightward shift in HLA-DR signal intensity within the CD11c^+^ population, suggesting activation-associated changes in HLA-DR expression of incorporated myeloid/APC-like cells under allergen-induced inflammatory conditions (Fig. 6f).

HDM-treated VSAs secreted higher levels of AD-associated inflammatory mediators (TSLP, CCL17/TARC, IL-4, IL-13), together with vascular inflammatory mediators (VEGF, NO), as measured by ELISA (Fig. 6g–l). Together, these findings indicate that HDM stimulation induces inflammatory remodelling in immune-integrated VSAs with AD-relevant features, characterized by barrier perturbation, epidermal hyperplasia-like remodelling, immune-cell infiltration, myeloid/APC-like activation-associated changes and type 2-associated inflammatory mediator production.

### Clinically approved atopic dermatitis drugs attenuate house dust mite-induced inflammatory responses in vascularized skin assembloids

**Fig. 7:**
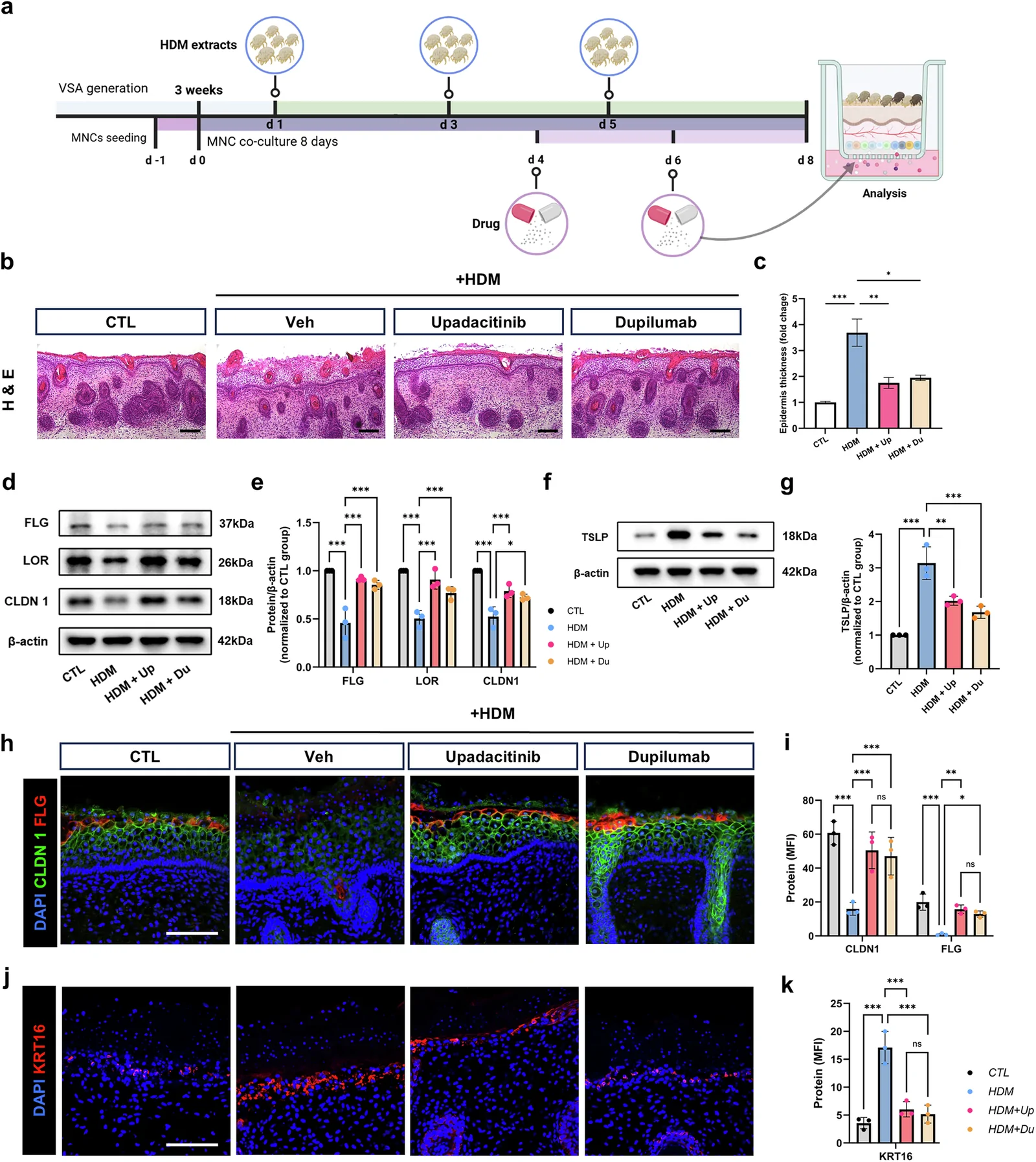
Pharmacological modulation of house dust mite-induced inflammatory responses in vascularized skin assembloids. **a** Schematic illustration of upadacitinib (Up) and dupilumab (Du) treatment in house dust mite (HDM)-induced vascular skin assembloids (VSAs). **b**, **c** Haematoxylin and eosin (H & E) staining and quantification showing reduction of HDM-induced epidermal hyperplasia after drug treatment (*n* = 3). **d**, **e** Western blot and quantification of FLG, LOR and CLDN1 expression, showing improved barrier-associated marker expression. **f**, **g** Western blot and quantification of TSLP expression demonstrating suppression of inflammatory mediators (*n* = 3). **h–k** Immunofluorescence staining and quantification showing increased filaggrin (FLG) (red) and CLDN1 (green) expression and suppression of KRT16 (red) after Up or Du treatment. All scale bars, 100 μm. Data are presented as mean ± standard deviation. Statistical significance was determined by one-way analysis of variance with Tukey’s post hoc test (*P < 0.05, **P < 0.01, ***P < 0.001). Figure 7a was created with BioRender.com. CTL, control; MFI, mean fluorescence intensity; MNC, mononuclear cell; ns, not significant.

To assess the pharmacological responsiveness of the HDM-induced inflammatory phenotype, we tested two clinically approved AD drugs with distinct mechanisms of action (Fig. 7a). Upadacitinib, a JAK inhibitor with proven efficacy in moderate-to-severe AD^30,31^, and dupilumab, an IL-4Rα antagonist that blocks IL-4/IL-13 signalling^32,33^, were selected. Dose–response analyses determined the optimal concentrations of each agent using *FLG* as a representative barrier marker and *TARC/TSLP* as Th2-associated mediators. Upadacitinib increased *FLG* expression in a concentration-dependent manner up to 1 μM, whereas higher concentrations reduced efficacy and induced rebound increases in *TARC/TSLP* (Supplementary Fig. 6a). Dupilumab also increased FLG expression and reduced *TARC/TSLP* levels, with responses approaching saturation above 10 μg ml^−1^ without further benefit (Supplementary Fig. 6b). Drug concentrations were therefore chosen to maximize barrier-associated marker recovery while reducing inflammatory mediator production. Histological analyses confirmed drug-associated attenuation of the HDM-induced phenotype. HDM-treated VSAs exhibited pronounced epidermal hyperplasia, which was significantly reduced by upadacitinib or dupilumab, shifting epidermal thickness towards control levels (Fig. 7b, c). Protein analysis showed increased FLG, LOR and CLDN1 expression with concomitant suppression of TSLP (Fig. 7d–g). Immunofluorescence showed that upadacitinib or dupilumab increased FLG and CLDN1 expression while suppressing KRT16 induction compared with HDM-treated VSAs (Fig. 7h–k). Bulk RNA-seq of upadacitinib-treated VSAs corroborated these outcomes, displaying transcriptional patterns consistent with improved barrier-associated marker expression (Supplementary Fig. 7). Collectively, these results demonstrate that upadacitinib and dupilumab attenuate HDM-induced inflammatory responses in VSAs by improving barrier-associated marker expression and reducing type 2-associated inflammatory mediators. The observed pharmacological responses are consistent with the known mechanisms of clinically used AD therapeutics and support the utility of the VSA platform for preclinical pharmacological evaluation of anti-inflammatory and barrier-modulating agents.

### House dust mite–vascularized skin assembloid model shows STAT6-associated inflammatory signalling and pharmacological modulation

**Fig. 8:**
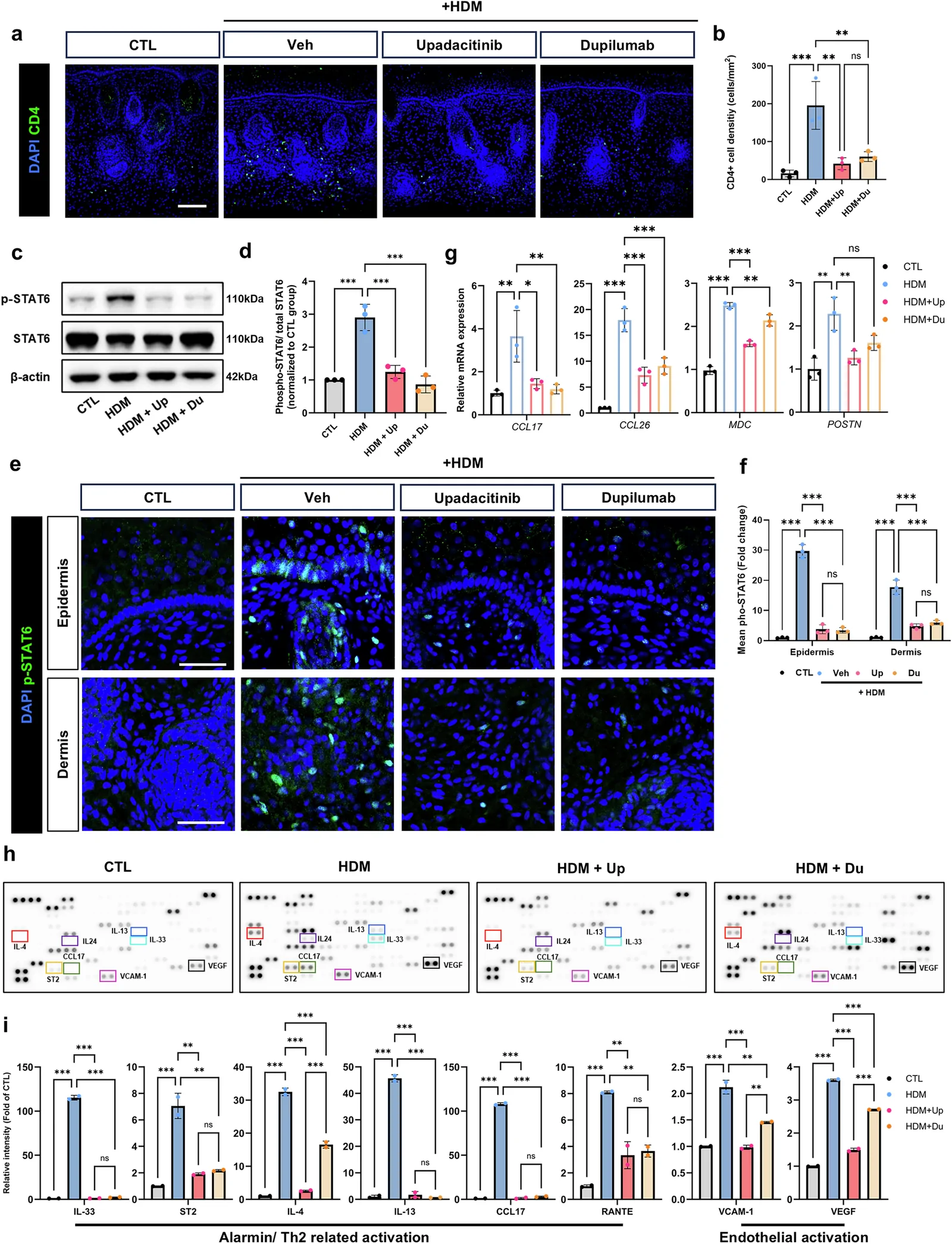
Pharmacological modulation of STAT6-associated inflammatory signalling in house dust mite-treated vascularized skin assembloids. **a** Representative immunofluorescence images of CD4^+^ T cells (green) showing accumulation in the upper dermis of house dust mite (HDM)-treated vascularized skin assembloids (VSAs) and reduced accumulation after upadacitinib (Up) or dupilumab (Du) treatment. **b** Quantification of CD4^+^ T cell density within the upper dermis (*n* = 3). **c**, **d** Western blot analysis and quantification of p-STAT6/STAT6 ratios using whole-VSA lysates, showing that HDM-induced STAT6 phosphorylation was reduced by Up and Du, with total STAT6 unchanged. **e**, **f** Immunofluorescence and quantification of nuclear p-STAT6 showing reduced tissue-level p-STAT6 signal after treatment. **g** Quantitative real-time PCR analysis of STAT6-associated inflammatory genes (*TARC*, *MDC*, *CCL26*, *POSTN*) showing attenuation following treatment. **h**, **i** Cytokine array profiling and quantification showing suppression of HDM-induced inflammatory mediators (IL-33, ST2, IL-4, IL-13, CCL17, RANTES, VCAM1, VEGF) after treatment. All scale bars, 100 μm. Data are presented as mean ± standard deviation. Statistical significance was determined by one-way analysis of variance with Tukey’s post hoc test (*P < 0.05, **P < 0.01, ***P < 0.001). CTL, control; ns, not significant; Veh, vehicle.

To further define how pharmacological agents modulate HDM-induced inflammatory responses, we investigated immune-cell infiltration, STAT6 activity and cytokine signalling within the HDM–VSA model. In HDM-treated VSAs, CD4^+^ T cells accumulated predominantly in the upper dermis, consistent with enhanced immune-cell infiltration under HDM-induced inflammatory conditions. By contrast, VSAs treated with Up or Du displayed markedly reduced infiltration in this region, with immune cells largely confined to the lower dermis (Fig. 8a, b). This redistribution suggests that both agents attenuate HDM-induced immune-cell infiltration and alter the spatial distribution of infiltrating immune cells within VSAs. These findings indicate that the VSA model can capture tissue-level immune modulation within a skin-like tissue context. HDM stimulation markedly increased phosphorylation of STAT6, a central mediator of IL-4/IL-13-driven type 2 inflammatory signalling, whereas total STAT6 levels remained unchanged (Fig. 8c, d). Treatment with either upadacitinib or dupilumab significantly decreased p-STAT6 abundance, indicating inhibition of STAT6 pathway activation. Immunofluorescence confirmed these findings at the tissue level, showing nuclear translocation of p-STAT6 in both epidermal and dermal compartments of HDM-treated VSAs, which was markedly diminished following drug treatment (Fig. 8e, f). These results suggest that JAK1 inhibition and IL-4Rα antagonism both lead to suppression of STAT6 activation^34,35^, thereby reducing downstream type 2-associated inflammatory signalling within the HDM–VSA model. Expression of STAT6-associated target genes, including *TARC*, *MDC*, *CCL26* and *POSTN*, was strongly induced by HDM but attenuated by both agents (Fig. 8g). Furthermore, HDM exposure elevated multiple AD-associated cytokines and chemokines such as IL-33, ST2, IL-4, IL-13, CCL17, RANTES, VCAM1 and VEGF, reflecting broad immune activation across epithelial and vascular compartments (Fig. 8h, i). The parallel reduction of epithelial alarmins, type 2-associated cytokines and vascular activation markers following treatment indicated the broad immunomodulatory effects of both agents. Transcriptomic data from upadacitinib-treated VSAs supported these findings, showing concurrent downregulation of type 2-associated cytokine genes and improvement of barrier-related programmes (Supplementary Fig. 7). Together, these findings suggest that HDM stimulation induces STAT6-associated type 2 inflammatory signalling, which is mitigated by upadacitinib and dupilumab. The HDM–VSA model therefore provides a useful framework for evaluating mechanism-consistent pharmacological responses in a skin-like inflammatory context.

## Discussion

Human skin constitutes a highly complex multicellular ecosystem that integrates epithelial, stromal, vascular and immune compartments to maintain barrier integrity and tissue homeostasis^36,37^. Disruption of this intricate system underlies a wide spectrum of inflammatory and degenerative skin disorders that collectively impose major clinical and socioeconomic burdens. Yet, mechanistic understanding and therapeutic development remain constrained by the lack of physiologically relevant human models that integrate epithelial, vascular and immune compartments. Reconstructing human skin in vitro has long been recognized as a critical benchmark in tissue modelling, as it requires coordinated multicellular organization that reflects aspects of native barrier, stromal and vascular–immune interactions. Although reconstructed skin equivalents and organoid models have advanced our understanding of epidermal differentiation, they lack integrated vascular and immune-associated compartments, limiting their capacity to model dynamic inflammatory processes^38,39^. Animal models, though valuable for systemic analyses, remain constrained by interspecies differences in cytokine networks, immune repertoires and epidermal structure, thereby limiting translational relevance^10^.

To overcome these limitations, we established VSAs by modularly integrating hiPSC-derived SkOs and BVOs under ALI conditions. This assembly strategy enabled spatially controlled fusion of multiple lineage-specific tissue modules, supporting the formation of a skin-like tissue organization, including a stratified epidermis, fibroblast-enriched dermal compartment and interconnected vascular-like networks. The conceptual advance of this platform lies not only in incorporating vascular and immune-associated components but also in establishing a modular and experimentally accessible skin assembloid architecture. Unlike conventional self-organized organoids, the modular integration approach allows separately generated epithelial and vascular tissue modules to be assembled, matured under ALI conditions and subsequently subjected to defined immune-cell incorporation, epidermal-side allergen exposure and pharmacological perturbation. This design provides experimental control over the timing and composition of modular tissue assembly, immune-cell incorporation, and inflammatory or pharmacological perturbation. The resulting vascular networks supported dermal organization and epidermal maturation and provided a structural basis for immune-cell localization and localized cytokine responses. Thus, VSAs offer a complementary strategy to recently reported vascularized or immune-associated skin organoid systems by enabling controlled inflammatory modelling and pharmacological testing within a compartmentally accessible skin-like tissue context. Collectively, these features support the utility of VSAs as an experimentally tunable skin assembloid platform for modelling vascular- and immune-associated inflammatory responses.

An important feature of the VSA system is its ability to assess vascular-associated inflammatory readouts, which are relevant to inflammatory skin pathophysiology^40–42^. Upon exposure to LPS, VSAs exhibited upregulation of endothelial adhesion molecules (ICAM1, VCAM1) and pro-inflammatory cytokines (IL-6, TNF-α), accompanied by MNC localization near CD31^+^ vascular structures within the dermal compartment. These responses were diminished in avascular SkOs, suggesting that vascularization supports immune-cell localization and endothelial inflammatory activation in the VSA platform^43^. Together, these findings support the potential application of VSAs for studying selected vascular-associated inflammatory responses in vitro. Although VSAs displayed vascular-like networks with endothelial junctions, basement membrane deposition, perivascular association and partial arterial- and venous-associated marker expression, these features should be interpreted as vascular organization within the current static ALI culture system rather than complete vascular hierarchy maturation.

To further evaluate its disease relevance, VSAs were challenged with HDM extract, a clinically relevant allergen that triggers type 2-associated inflammatory responses in human skin^44^. HDM exposure induced pronounced epidermal hyperplasia, reduced expression of the barrier protein FLG and elevated KRT16 expression, indicative of keratinocyte stress and barrier dysfunction. Transcriptomic analyses revealed global activation of inflammatory, keratinization and cytokine signalling pathways, accompanied by marked upregulation of type 2/alarmin-associated mediators (*IL33*, *IL4R*, *IL13RA2*, *CCL17*, *CCL26*, *GATA3*) and epithelial stress markers (*KRT16*, *SPRR2A/B*, *S100A8/9*, *POSTN*). Comparative analysis with published AD patient skin transcriptomic data further showed that HDM-treated VSAs partially paralleled AD-associated molecular signatures, particularly type 2/alarmin activation, keratinocyte activation/stress responses and adhesion/infiltration-associated programmes. Immune cells accumulated within the dermal compartment and localized near vascular structures, suggesting that the integrated vasculature contributes to spatially organized inflammatory readouts in the VSA platform^45^. Collectively, these findings demonstrate that VSAs exhibit selected molecular and tissue-level features of HDM-induced inflammatory remodelling with relevance to AD, including epithelial stress, barrier perturbation, immune-cell infiltration and vascular-associated inflammatory readouts. The pharmacological responsiveness of the VSA platform was evaluated using clinically approved AD therapeutics. Treatment with upadacitinib (a JAK1 inhibitor) or dupilumab (an IL-4Rα antagonist) improved barrier-associated marker expression by upregulating differentiation and junctional markers (FLG, LOR, CLDN1) and suppressing inflammatory mediators such as TSLP and KRT16. Transcriptomic profiling of upadacitinib-treated VSAs supported these findings, revealing coordinated downregulation of type 2-associated cytokine networks (*IL4R*, *IL13RA1*, *TSLP*, *CCL17*, *CCL22*) and modulation of epidermal differentiation and extracellular matrix-related pathways, consistent with modulation of the JAK–STAT6 axis. Both drugs were associated with reduced STAT6 phosphorylation and STAT6-associated inflammatory target gene expression. These pharmacological responses are mechanistically consistent with the known mechanisms of these therapeutics, whereas the current model does not yet capture inter-patient variability in pharmacological responses or the full chronicity of clinical AD^46–48^.

Despite these advances, several limitations remain. Although VSAs contain interconnected vascular-like networks, they do not yet reproduce a fully mature perfusable vascular hierarchy. Although VSAs displayed endothelial junctions, basement membrane deposition, perivascular association and partial arterial- and venous-associated marker expression, the current static ALI culture system lacks sustained blood flow and haemodynamic cues required for mature artery–vein–capillary hierarchy formation. Thus, these findings should be interpreted as evidence of vascular organization and arterial-/venous-associated endothelial features rather than complete vascular hierarchy maturation^49^. Future incorporation of microfluidic perfusion systems or organ-on-a-chip platforms could provide controlled haemodynamic cues, continuous nutrient exchange and real-time monitoring of vascular dynamics, thereby enhancing vessel maturation and stability^50–52^. In addition, definitive lymphatic vessel-like structures were not observed under the present culture conditions. Because lymphatic vessels contribute to fluid drainage, antigen transport and immune-cell trafficking in skin inflammation, incorporation of lymphatic endothelial components may further improve the physiological fidelity of the platform.

The immune component, derived from umbilical cord blood mononuclear cells, provides an experimentally tractable immune-integrated system but introduces donor variability and does not fully recapitulate native skin-resident immunity. In particular, the current VSA model lacks bona fide Langerhans cells, tissue-resident memory T cells, and functionally specialized skin dendritic cell subsets^53^. Flow cytometric analysis identified CD45^+^ immune cells, T cell subsets, CD14^+^ myeloid-lineage cells, and CD11c^+^HLA-DR^+^ or CD11c^+^CD1c^+^ APC-like populations within VSA–MNC co-cultures. However, these populations should be interpreted as MNC-derived myeloid/APC-like immune cells based on marker expression, rather than as definitive skin-resident antigen-presenting cells or functionally mature dendritic cells. Future incorporation of hiPSC-derived or patient-specific immune lineages, including Langerhans-like cells, dendritic cells, macrophages and tissue-resident memory T cells, could allow more controlled modelling of antigen presentation, adaptive immune specialization and patient-specific inflammatory responses. Moreover, vascular–immune interactions were primarily inferred from histological proximity, flow cytometry and gene-expression patterns; spatial transcriptomics and single-cell multiomics approaches will be valuable for elucidating cell-type-specific crosstalk at single-cell resolution^54,55^.

Another important consideration is that the present HDM–VSA model reflects an acute allergen-induced inflammatory response rather than a fully chronic AD model. Human AD involves recurrent barrier disruption, prolonged type 2-associated inflammation, tissue remodelling, microbial dysbiosis and substantial patient-to-patient heterogeneity. Although HDM-treated VSAs exhibited selected AD-associated features, including epidermal remodelling, type 2/alarmin-associated molecular signatures, immune-cell infiltration and pharmacological responsiveness to clinically used agents, the current platform does not yet model long-term disease recurrence or chronic inflammatory memory. Future studies incorporating prolonged or repeated allergen exposure, disease-relevant microbial components, patient-derived hiPSC lines and defined resident immune populations may enable more refined modelling of chronic AD-associated processes and individualized pharmacological responses.

Beyond inflammatory modelling, the structural and functional attributes of VSAs suggest potential future applications in regenerative skin research. The preformed vascular-like network, stabilized by perivascular cells and surrounded by basement membrane components, may support host–graft vascular association and tissue maintenance after transplantation, a recognized challenge in autologous and bioengineered skin grafts. Additionally, integrating VSAs with biomaterial scaffolds, bioprinting technologies or microfluidic perfusion systems could extend their translational relevance for wound healing, vascular regeneration and reconstructive dermatology^56–58^. In this context, the modular nature of the VSA system may be particularly useful for testing bioactive materials that independently modulate epidermal maturation, vascular stabilization, immune-cell recruitment or inflammatory resolution.

From a broader perspective, VSAs represent a modular, human cell-derived platform that links mechanistic studies with translationally oriented applications. By capturing barrier-associated changes, immune-associated activation, vascular organization and pharmacologically responsive inflammatory readouts within a human-relevant context, this model provides a useful testbed for studying inflammatory mechanisms, evaluating pharmacological agents that modulate inflammatory signalling, and assessing bioactive materials designed to modulate skin regeneration or inflammation. In conclusion, VSAs provide a modular experimental framework that integrates epithelial, vascular and immune-associated components in a reproducible and tunable system, supporting mechanistic studies and controlled preclinical applications in inflammatory skin disease modelling and biomaterials evaluation.

## Data and materials availability

The raw sequencing data generated in this study have been deposited in the Sequence Read Archive under the BioProject accession code PRJNA1339004.

## Supplementary information

Supplementary Information
