# Supplementary material for: Vascularized human skin assembloids model compartmental skin organization and immune responsiveness in vitro
Source: Exp Mol Med. 2026 Oct 1;58(10):3339–56. doi: 10.1038/s12276-026-01840-x (PMC13737209; doi:10.1038/s12276-026-01840-x)
Supplement: Supplementary file 1 [file 12276_2026_1840_MOESM1_ESM.pdf]

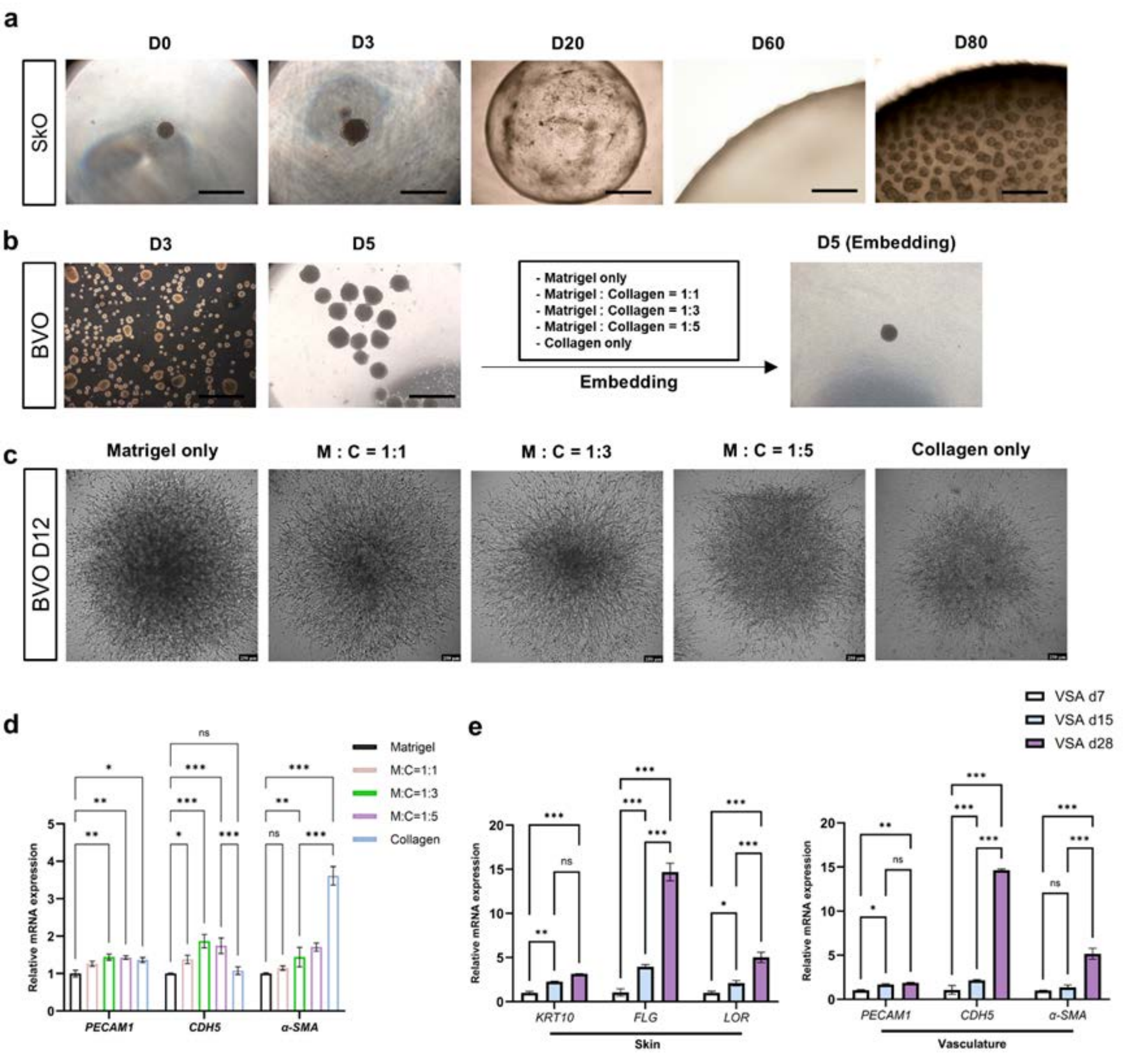

Supplementary Figure 1. Optimization of extracellular matrix composition and temporal maturation of VSAs.

(a) Representative bright-field images of hiPSC-derived skin organoids (SkOs) differentiation from day 0 to day 80. Scale bars, 100 μm.

(b) Generation of blood vessel organoids (BVOs) from mesodermal aggregates at day 3 and day 5, followed by embedding into Matrigel–collagen (M:C) matrices of varying ratios (Matrigel only, 1:1, 1:3, 1:5, or collagen only). Representative image of BVO embedding at D5 is shown. Scale bars, 100 μm.

(c) Representative bright-field images of BVOs cultured under different ECM conditions at day 12. (d) Quantitative PCR analysis of endothelial and mural cell markers (*PECAM1*, *CDH5*, *α-SMA*) in BVOs cultured under the indicated ECM conditions (n = 3).

(e) Temporal gene expression profiling of VSAs at D7, D15, and D28 showing epidermal differentiation markers (*KRT10*, *FLG*, *LOR*) and vascular markers (*PECAM1*, *CDH5*), along with α-SMA (n = 3). Data

are presented as mean ± SD. Statistical differences were determined using one-way ANOVA followed by Tukey's post hoc test (ns, not significant; *P < 0.05; **P < 0.01; ***P < 0.001).

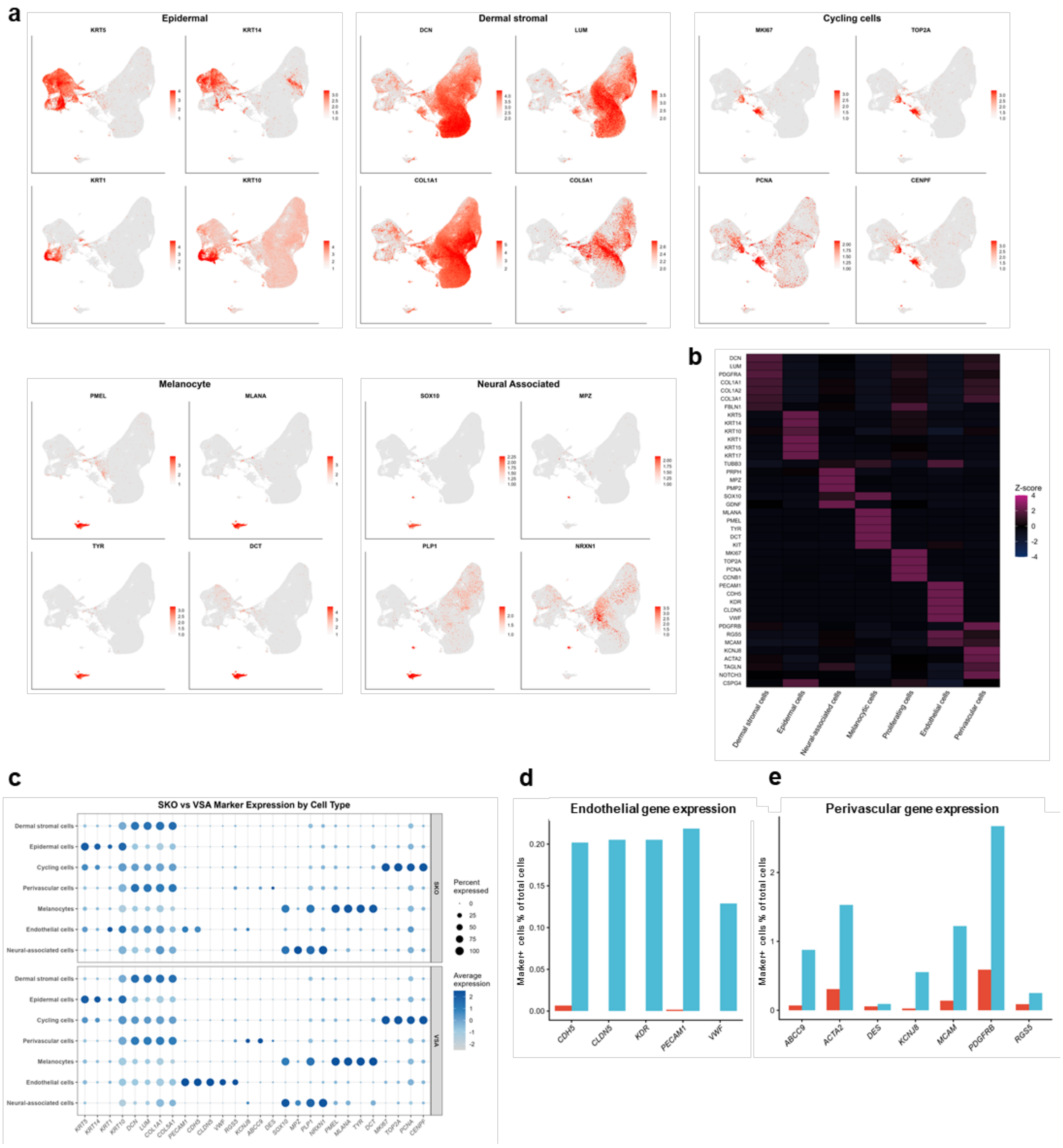

Supplementary Figure 2. Extended single-cell transcriptomic profiling of VSAs.

(a) UMAP feature plots showing the expression of representative cell population-associated markers, including epidermal markers *KRT5*, *KRT14*, *KRT1*, and *KRT10*, dermal stromal markers *DCN*, *LUM*, *COL1A1*, and *COL5A1*, cycling cell markers *MKI67*, *TOP2A*, *PCNA*, and *CENPF*, melanocyte markers *PMEL*, *MLANA*, *TYR*, and *DCT*, and neural-associated markers *SOX10*, *MPZ*, *PLP1*, and *NRXN1*.

(b) Heatmap showing scaled expression of selected marker genes across annotated cell populations, including dermal stromal, epidermal, neural-associated, melanocytic, proliferative, endothelial, and perivascular cells.

(c) Dot plot comparing marker gene expression patterns between SkOs and VSAs across annotated cell populations, supporting the annotation of major skin-associated epithelial, stromal, neural, melanocytic, proliferative, endothelial, and perivascular populations. Dot size represents the percentage of marker-expressing cells, and color intensity indicates average scaled expression.

(d, e) Quantification of cells expressing endothelial marker genes *CDH5*, *CLDN5*, *KDR*, *PECAM1*, and *VWF* (d) and perivascular marker genes *ABCC9*, *ACTA2*, *DES*, *KCNJ8*, *MCAM*, *PDGFRB*, and *RGS5* (e) in SkOs and VSAs, shown as the percentage of total cells.

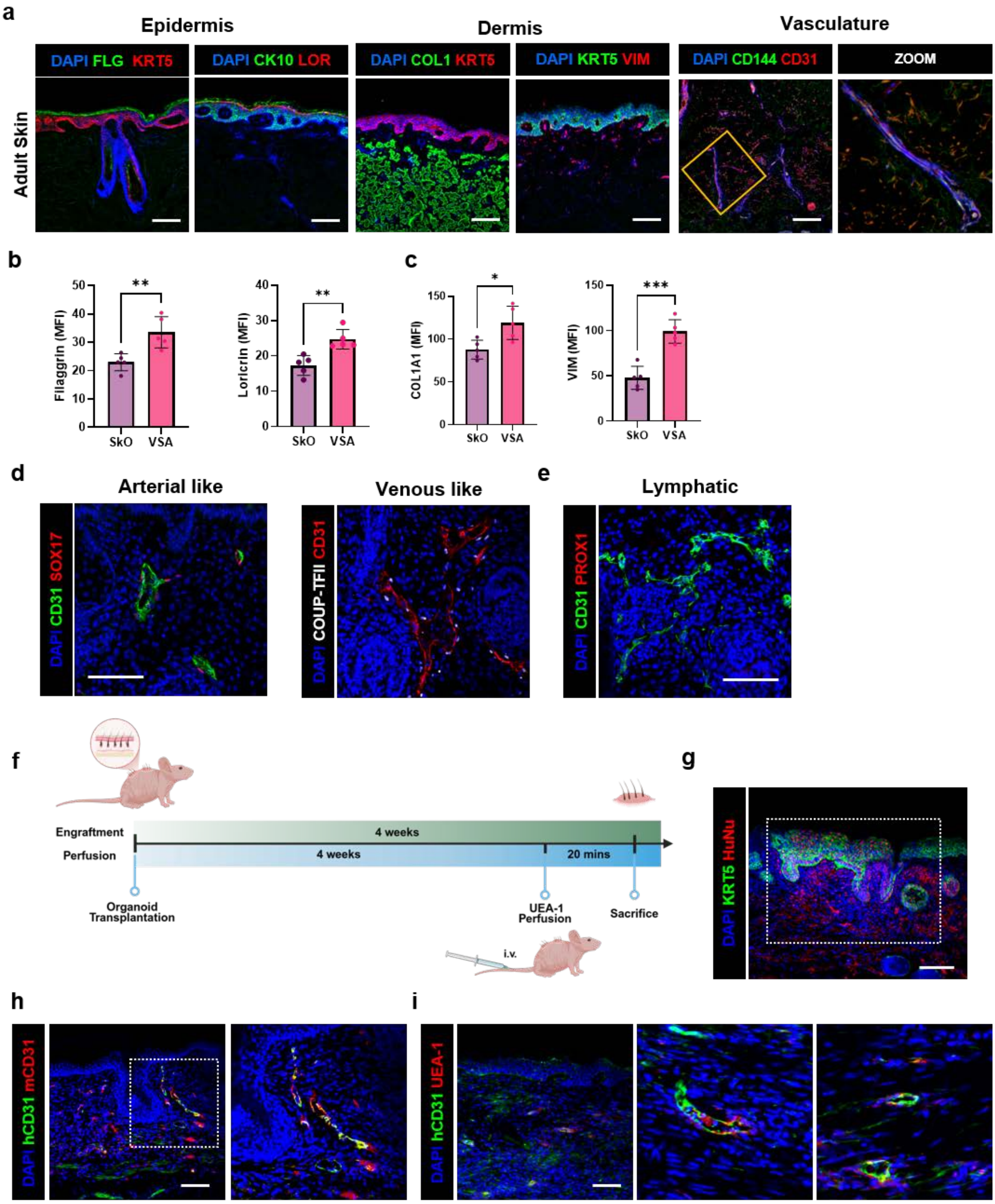

Supplementary Figure 3. Structural characterization, vascular identity, and in vivo perfusion analysis of VSAs.

(a) Representative immunofluorescence images showing the expression of epidermal and dermal structural markers in VSAs, including FLG/KRT5, CK10/LOR, COL1/KRT5, and KRT5/VIM. Immunostaining for CD144 and CD31 further demonstrates the presence of vascular structures, and the boxed region is shown at higher magnification in the zoomed image.

(b,c) Quantification of FLG, LOR, COL1, and VIM expression in SkOs and VSAs. Data are presented as mean ± SD. Statistical significance was determined using an unpaired Student's t-test (*$P < 0.05$, **$P < 0.01$, ***$P < 0.001$).

(d,e) Immunofluorescence staining of vascular subtype-associated markers showing CD31/SOX17, COUP-TFII/CD31, and CD31/PROX1 in VSAs, indicating arterial- and venous-associated endothelial features and the absence of clearly organized PROX1^{+} lymphatic vessel-like structures under the current culture conditions.

(f) Schematic illustration of the in vivo transplantation and perfusion assay timeline. VSAs were transplanted and maintained for 4 weeks, followed by UEA-1 injection 20 min before tissue harvest for vascular perfusion analysis. Schematic illustration was created with BioRender.com.

(g) Immunofluorescence image of the grafted tissue stained for KRT5 and human nuclei (HuNu), showing the engraftment and maintenance of the human epidermal component in vivo.

(h) Immunofluorescence images stained for human CD31 (hCD31) and mouse CD31 (mCD31), showing the association between graft-derived human vessels and host vasculature, with a magnified view of the boxed region.

(i) Immunofluorescence images stained for hCD31 and UEA-1, demonstrating in vivo perfusion of graft-associated vascular structures, with higher-magnification images shown on the right. Scale bars, as indicated.

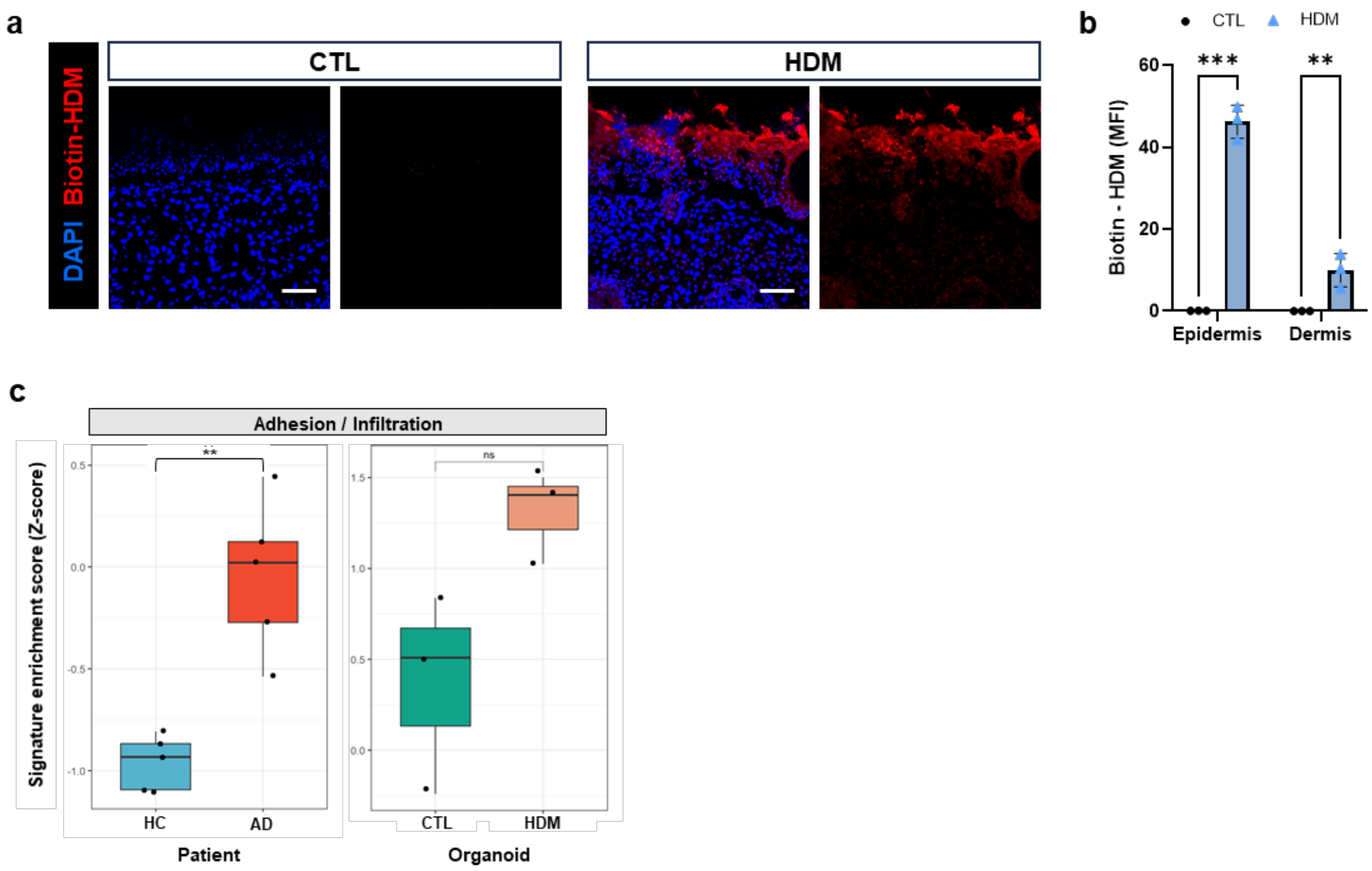

Supplementary Figure 4. HDM penetrates VSAs and induces adhesion/infiltration-associated molecular features.

(a) Representative fluorescence images showing penetration of biotin-labeled HDM extract (red) in control (CTL) and HDM-treated VSAs. Biotin-HDM signal was detected in both the epidermal and dermal compartments following HDM treatment. Nuclei were counterstained with DAPI (blue). Scale bars, 100 μm.

(b) Quantification of biotin-HDM mean fluorescence intensity (MFI) in the epidermis and dermis of CTL and HDM-treated VSAs. Data are presented as mean ± SD. Statistical significance was determined using an unpaired Student's t-test (***P* < 0.01, ****P* < 0.001).

(c) Box plot showing adhesion/infiltration-related gene signature enrichment scores (Z-scores) in patient samples (HC vs AD) and organoid samples (CTL vs HDM). These data indicate that HDM-treated VSAs exhibit increased adhesion/infiltration-associated transcriptional features that partially parallel those observed in AD patient skin samples. Statistical significance was determined using a two-tailed unpaired Student's t-test for comparisons between two groups within each dataset.

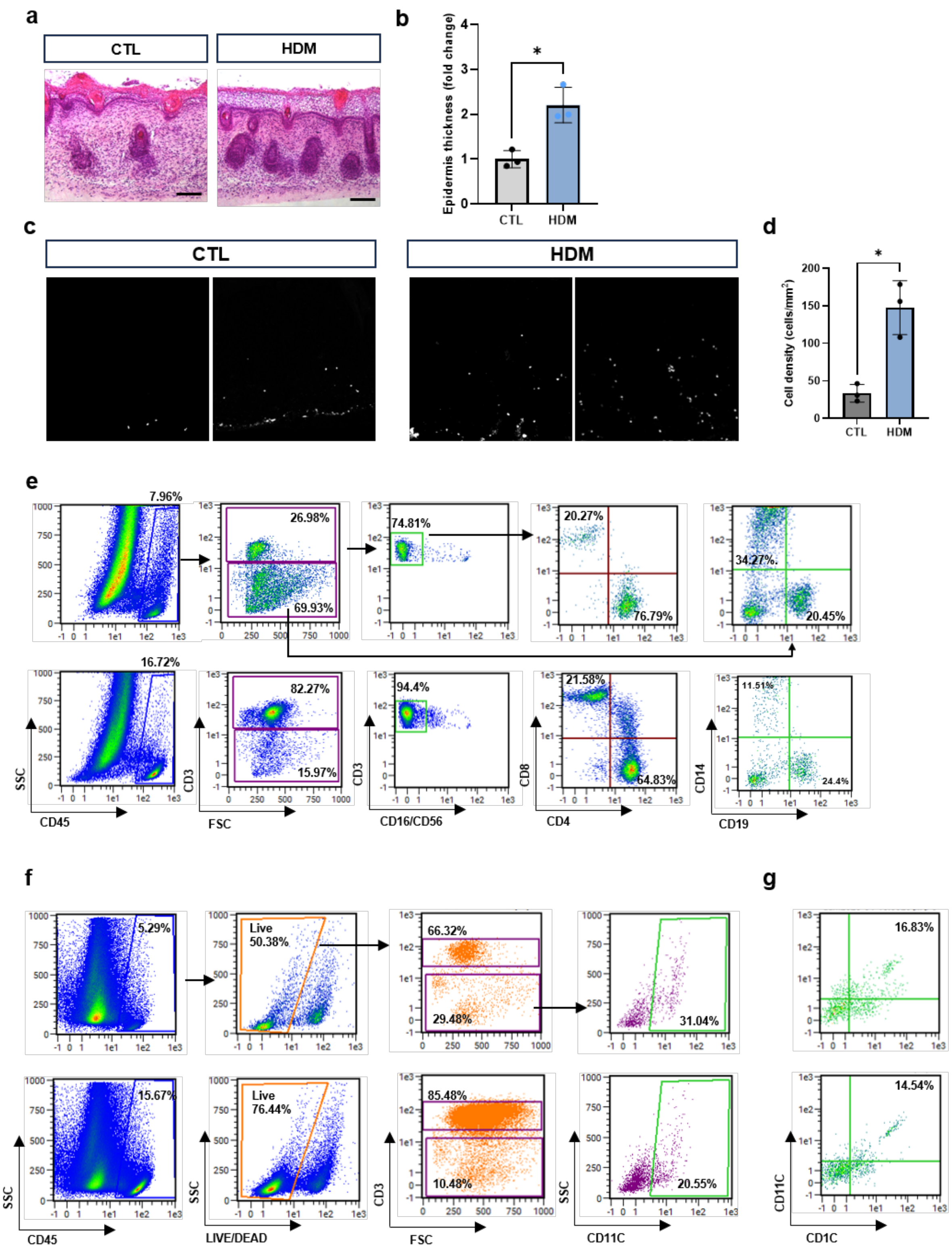

Supplementary Figure 5. Additional characterization of HDM-induced tissue remodeling and immune-cell composition in VSAs.

(a) Representative H&E-stained images of CTL and HDM-treated VSAs showing epidermal thickening and tissue remodeling after HDM stimulation.

(b) Quantification of epidermal thickness in CTL and HDM-treated VSAs.

(c, d) Representative fluorescence images and quantification showing increased tissue-associated signal in HDM-treated VSAs compared with CTL, indicating enhanced penetration and/or permeability-associated changes following HDM exposure.

(e–g) Representative flow cytometry plots showing the gating strategy and immune-cell profiling of CTL and HDM-treated VSAs. Recovered cells were analyzed for $CD3^+$ T-cell populations and $CD3^-$ myeloid/APC-associated populations expressing CD11c, HLA-DR, and CD1c.

Scale bars, 100 μm. Data are presented as mean ± SD. Statistical significance was determined using an unpaired Student's t-test (*$P < 0.05$)

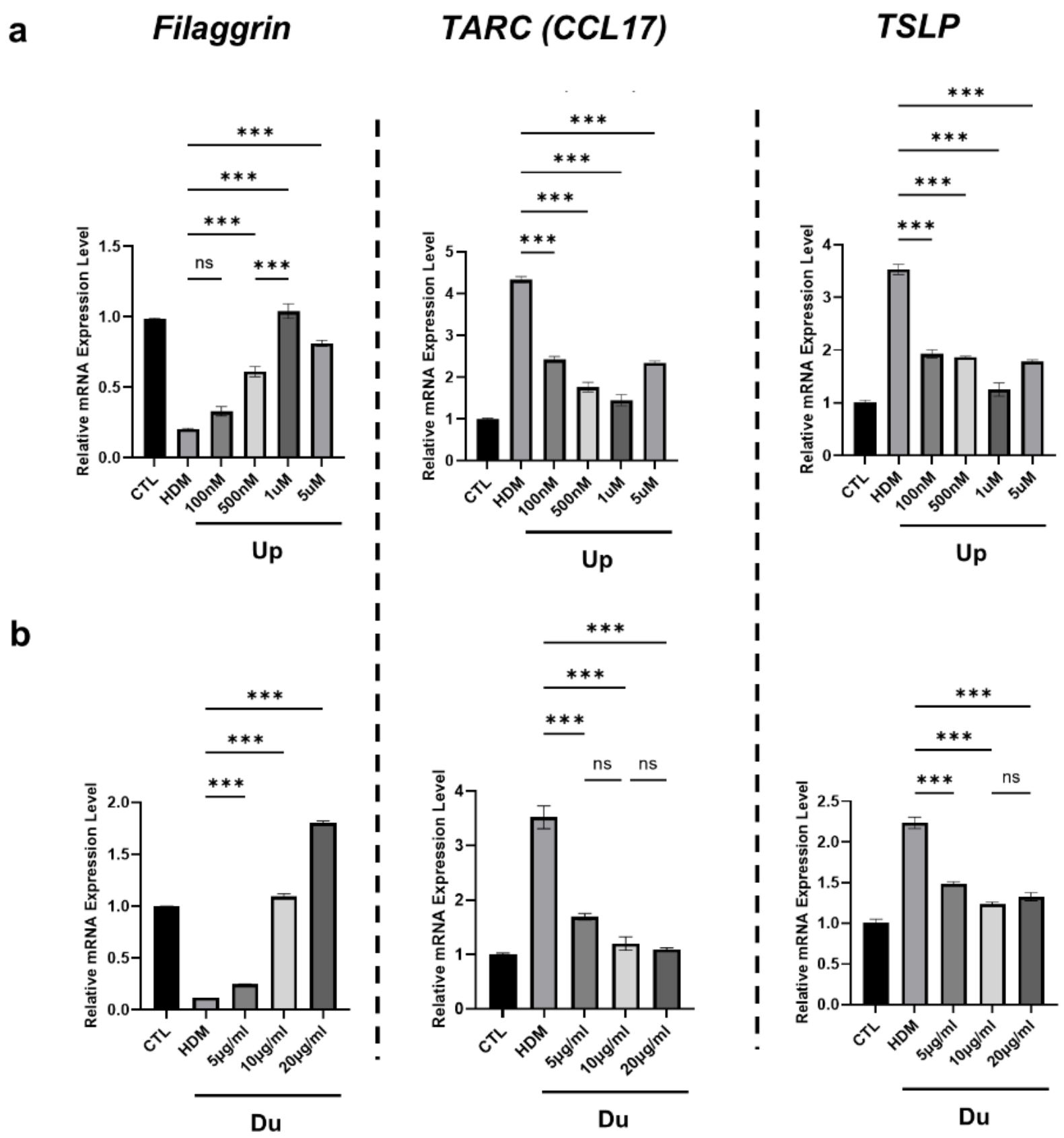

Supplementary Figure. 6. Dose–response optimization of Upadacitinib and Dupilumab in HDM-treated VSAs.

(a) Dose–response analysis of Upadacitinib (Up; 0.1–5 μM) in HDM-treated VSAs. qPCR analysis of the barrier-associated marker *FLG* and type 2/inflammatory mediators (*TARC*, *TSLP*) showing increased *FLG* expression and reduced inflammatory mediator expression at the selected working concentration (1 μM).

(b) Dose–response analysis of Dupilumab (Du; 5–20 μg mL^{-1}) in HDM-treated VSAs. Du increased barrier-associated marker expression, with reduced *TARC/TSLP* expression observed at concentrations above 10 μg mL^{-1}.

Data are presented as mean ± SD. Statistical difference was determined using one-way ANOVA followed by Tukey's post hoc test (ns, not significant; *$P < 0.05$, **$P < 0.01$, ***$P < 0.001$).

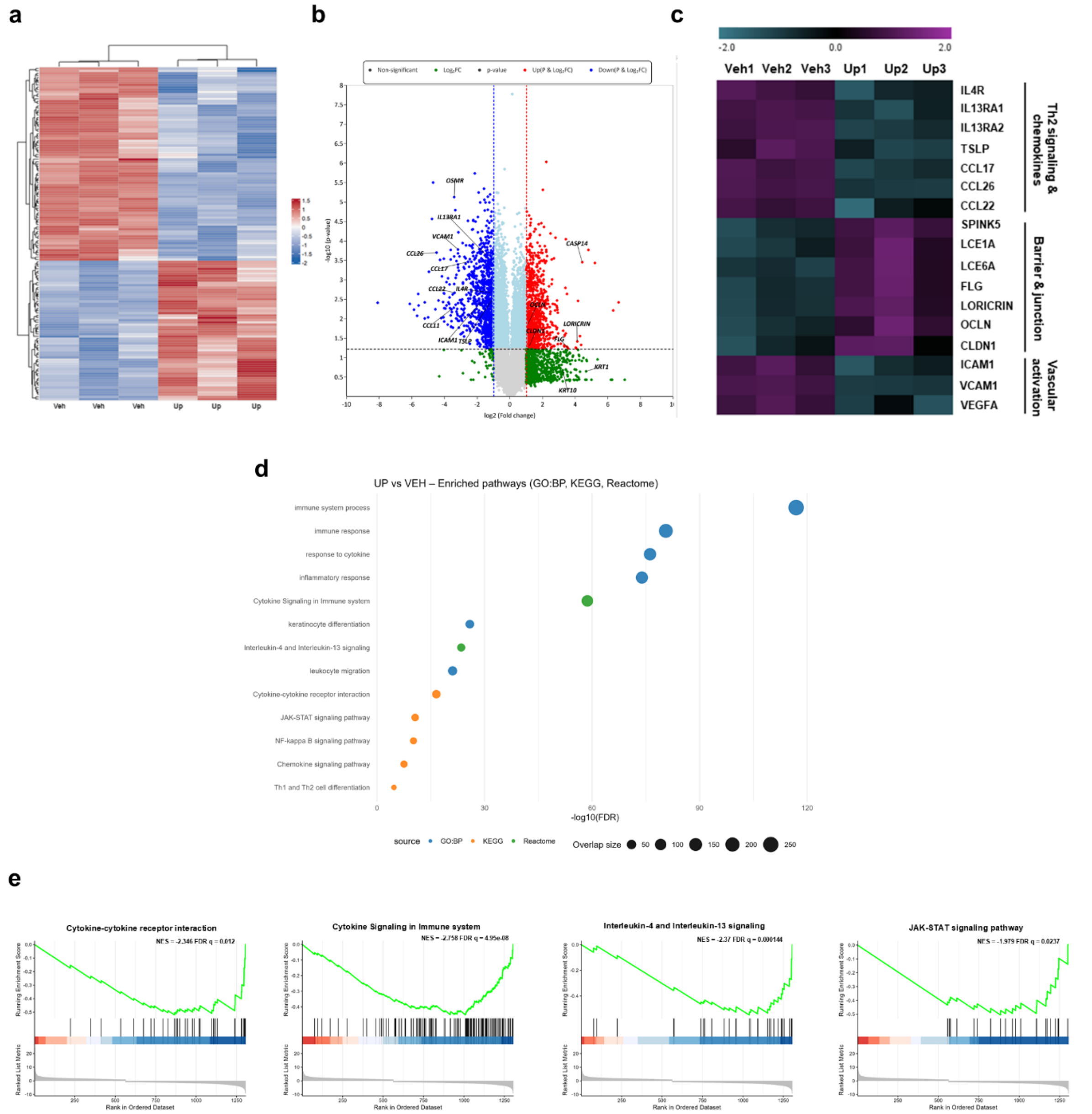

Supplementary Figure 7. Transcriptomic analysis of Up-treated VSAs reveals modulation of inflammatory signaling and barrier-associated programs.

(a) Hierarchical clustering heatmap of transcriptomic profiles comparing vehicle- and Up-treated VSAs, demonstrating distinct clustering.

(b) Volcano plot highlighting differentially expressed genes (DEGs) in Up-treated versus vehicle-treated VSAs.

(c) Heatmap of representative barrier-associated and type 2-associated inflammatory genes.

(d) Pathway enrichment analysis (GO:BP, KEGG, Reactome) of DEGs showing enrichment of cytokine signaling, inflammatory response, keratinocyte differentiation, and JAK–STAT-associated pathways.

(e) GSEA plots comparing Up-treated VSAs and vehicle-treated VSAs.

Supplementary Table 1. The list of antibodies used for immunofluorescence assay

Name	Source	Catalog
Mouse Anti-KRT14	Sigma-Aldrich	MAB3232
Rabbit Anti-CD31	Abcam	ab28364
Mouse Anti-Filaggrin (FLG)	Santa Cruz Biotechnology	Sc-66192
Rabbit Anti-KRT5	Abcam	ab52635
Mouse Anti-COL1A1 (COL1)	GeneTex	GTX26308
Mouse Anti-Vimentin (VIM)	Santa Cruz Biotechnology	Sc-6260
Mouse Anti-CD144	BD Pharmingen	555661
Mouse Anti-α-SMA	Abcam	ab7817
Rabbit Anti-COL4A1	Abcam	ab6586
Mouse Anti-ICAM-1	Santa Cruz Biotechnology	Sc-107
Rabbit Anti-KRT16	Abcam	ab76416
Rabbit Anti-CLDN1	Invitrogen	71-7800
Rabbit Anti-CD4	Abcam	ab183685
Rabbit Anti-Phospho-STAT6 (p-STAT6)	Cell Signaling Technology	9361
Mouse Anti-human nuclei	Millipore	MAB1281B
Mouse Anti-human CD31	Dako	M0823
Rat Anti-Mouse CD31	BD	5050274
PE-Streptavidin	eBioscience	12-4317-87

Supplementary Table 2. The list of antibodies used for Western blot

Name	Source	Catalog
Mouse Anti-β-actin	Abcam	ab8226
Rabbit Anti-Filaggrin (FLG)	Invitrogen	PA5116911
Rabbit Anti-Loricrin (LOR)	Abcam	ab85679
Rabbit Anti-CLDN1	Invitrogen	71-7800
Rabbit Anti-TSLP	Abcam	ab47943
Rabbit Anti-STAT6	Cell Signaling Technology	5397
Rabbit Anti-Phospho-STAT6 (p-STAT6)	Cell Signaling Technology	9361

Supplementary Table 3. The list of human primers used for qRT-PCR analysis

Human *GAPDH*	Forward	5′- GTC AGT GGT GGA CCT GAC CT -3′
	Reverse	5′- TGC TGT AGC CAA ATT CGT TG -3′
Human *ICAM1*	Forward	5′- GTG GTG GGG GAG ACA TAG CC -3′
	Reverse	5′- GGG GCC TCA GCA TAC CCA AT -3′
Human *VCAM1*	Forward	5′- GGT GAC GAA TGA GGG GAC CA -3′
	Reverse	5′- CCT CCA GAG GGC CAC TCA AA -3′
Human *IL-6*	Forward	5′- AAA GAT GTA GCC GCC CCA CA -3′
	Reverse	5′- GCC GTC GAG GAT GTA CCG AA -3′
Human *TNF-α*	Forward	5′- TTC CTT GAG ACA CGG AGC CC -3′
	Reverse	5′- GCT CCT ACA TTG GGT CCC CC -3′
Human *CCL17 (TARC)*	Forward	5′- ACC GCC TGC TGA TGG GAG A -3′
	Reverse	5′- CAG GAG GGA GTC TCT GTG TGC -3′
Human *CCL26*	Forward	5′- TGA GAG GCC GAG AAG TGG G -3′
	Reverse	5′- TTA TGA GAC TGA GAC GGC CCC -3′
Human *MDC*	Forward	5′- AGG AAG CTG GCT GTG GTA GC -3′
	Reverse	5′- TCT TTA GCC TGG CCC AAC CA -3′
Human *POSTN*	Forward	5′- TCA TTG ATG GAG TGC CTG TGG A -3′
	Reverse	5′- TCC TCA CGG GTG TGT CTT CT -3′
Human *PECAM1*	Forward	5′- GTG CTG CAA TGT GCT GTG AA -3′
	Reverse	5′- GCT TGG TCC AAA ATG CCT GG -3′
Human *CDH5*	Forward	5′- CTT CAC CCA GAC CAA GTA CAC A -3′
	Reverse	5′- AAT GGT GAA AGC GTC CTG GT -3′
Human *α-SMA*	Forward	5′- GAT CTG GCA CCA CTC TTT CTA C -3′
	Reverse	5′- CAG GCA ACT CGT AAC TCT TCT C -3′
Human *KRT10*	Forward	5′- TGC GTA GGG TGC TGG ATG AG -3′
	Reverse	5′- CCG GGG CAG CAT TCA TTT CC -3′
Human *Filaggrin (FLG)*	Forward	5′- TCG GCA AAT CCT GAA GAA TCC AGA -3′
	Reverse	5′- GCT TGA GCC AAC TTG AAT ACC ATC AG -3′
Human *LOR*	Forward	5′- CAC TCA CCC TTC CTG GTG CT -3′
	Reverse	5′- AGG GGT GGG CTG CTT TTT CT -3′
Human *TSLP*	Forward	5′- GAC TTG TGT TCC CCG CTC CT -3′
	Reverse	5′- CAA TGT GGC TGC TCA TAG GCG -3′
